# Half-calcified calmodulin promotes basal activity and inactivation of the L-type calcium channel Ca_V_1.2

**DOI:** 10.1016/j.jbc.2022.102701

**Published:** 2022-11-15

**Authors:** Peter Bartels, Ian Salveson, Andrea M. Coleman, David E. Anderson, Grace Jeng, Zoila M. Estrada-Tobar, Kwun Nok Mimi Man, Qinhong Yu, Elza Kuzmenkina, Madeline Nieves-Cintron, Manuel F. Navedo, Mary C. Horne, Johannes W. Hell, James B. Ames

**Affiliations:** 1 Department of Pharmacology, University of California, Davis, California, USA; 2 Department of Chemistry, University of California, Davis, California, USA; 3 Center for Pharmacology, University of Cologne, Cologne, Germany

**Keywords:** Ca_V_1.2, calmodulin, CDI, NMR, single-channel recordings, CaM, calmodulin, CDI, Ca^2+^-dependent inactivation, FP, fluorescence polarization, ITC, isothermal titration calorimetry, PVDF, polyvinylidene difluoride, RDC, residual dipolar coupling

## Abstract

The L-type Ca^2+^ channel Ca_V_1.2 controls gene expression, cardiac contraction, and neuronal activity. Calmodulin (CaM) governs Ca_V_1.2 open probability (Po) and Ca^2+^-dependent inactivation (CDI) but the mechanisms remain unclear. Here, we present electrophysiological data that identify a half Ca^2+^-saturated CaM species (Ca_2_/CaM) with Ca^2+^ bound solely at the third and fourth EF-hands (EF3 and EF4) under resting Ca^2+^ concentrations (50–100 nM) that constitutively preassociates with Ca_V_1.2 to promote Po and CDI. We also present an NMR structure of a complex between the Ca_V_1.2 IQ motif (residues 1644–1665) and Ca_2_/CaM_12’_, a calmodulin mutant in which Ca^2+^ binding to EF1 and EF2 is completely disabled. We found that the CaM_12’_ N-lobe does not interact with the IQ motif. The CaM_12’_ C-lobe bound two Ca^2+^ ions and formed close contacts with IQ residues I1654 and Y1657. I1654A and Y1657D mutations impaired CaM binding, CDI, and Po, as did disabling Ca^2+^ binding to EF3 and EF4 in the CaM_34_ mutant when compared to WT CaM. Accordingly, a previously unappreciated Ca_2_/CaM species promotes Ca_V_1.2 Po and CDI, identifying Ca_2_/CaM as an important mediator of Ca signaling.

Ca_V_1.2 is the main L-type channel in heart, blood vessels, and brain (1, 2). Ca^2+^ influx through Ca_V_1.2 triggers cardiac contraction, regulates arterial tone (1), mediates synaptic long-term potentiation (3, 4), controls neuronal excitability (5), and mediates Ca^2+^-dependent gene expression (6). Defects in inactivation of Ca_V_1.2 cause Timothy syndrome, a rare congenital abnormality leading to lethal arrhythmias, autistic-like behaviors, and immune deficiency (7). Thus, defining mechanisms of Ca_V_1.2 regulation is highly relevant for understanding its physiological and pathological functions. Ca^2+^ influx through Ca_V_1.2 triggers a rapid negative feedback mechanism by inducing channel inactivation called Ca^2+^-dependent inactivation (CDI) (8, 9). CDI is mediated by calmodulin (CaM) (8) that is preassociated with Ca_V_1.2 under basal Ca^2+^ conditions ([Ca^2+^]_i_ = 100 nM) (10, 11). Ca^2+^-free apoCaM has been suggested to be preassociated with Ca_V_1.2 (12) and the closely related Ca_V_1.3 (13). However, under physiological conditions, apoCaM binds to the isolated Ca_V_1.2 IQ-motif with a dissociation constant (*K*_*D*_) of ∼10 μM (14, 15) and ∼1 μM for full-length Ca_V_1.2 (11). The concentration of free apoCaM is <100 nM in neurons and cardiomyocytes (15, 16). Accordingly, the fractional binding of Ca_V_1.2 to apoCaM is predicted to be less than 10% and may not be the prevalent CaM species bound to Ca_V_1.2 or the closely related Ca_V_1.3 under basal conditions as proposed previously (12, 13, 17).

To fill a critical gap in our understanding of how CaM governs Ca_V_1.2 function, we used NMR structural analysis, protein biochemistry, and patch-clamp electrophysiology of WT and mutated Ca_V_1.2 bound to CaM. Our studies uncovered a half-calcified form of CaM (with Ca^2+^ bound solely at EF3 and EF4, called Ca_2_/CaM) that is functionally preassociated with Ca_V_1.2 under basal conditions. The NMR structure of Ca_2_/CaM bound to the Ca_V_1.2 IQ-motif (residues 1644–1664) suggests that the Ca^2+^-bound CaM C-lobe (residues F93, M110, L113, M125) forms intermolecular interactions with the side chain atoms from Ca_V_1.2 residues (Y1649, I1654, Y1657, and F1658), whereas the Ca^2+^-free CaM N-lobe does not interact with the IQ motif. Electrophysiological data of key mutants of Ca_V_1.2 (I1654A and Y1657E) contrasted with the earlier findings for the K1662E mutant along with the consequences of ectopic expression of CaM_34_ all suggest that Ca_2_/CaM, rather than apoCaM, preassociates with Ca_V_1.2 under basal conditions to augment channel open probability (Po) and mediate rapid CDI.

## Results

### A CaM intermediate with two Ca^2+^ bound

Isothermal titration calorimetry (ITC) studies have suggested that apoCaM binds to the IQ peptide with submicromolar affinity in the absence of salt (12). However, in the presence of physiological salt levels, apoCaM binds to the Ca_V_1.2 IQ-motif with a dissociation constant (*K*_*D*_) of 10 μM (14, 15). Earlier work suggests that binding of apoCaM to full-length Ca_V_1.2 is ∼10 times stronger than binding to the IQ segment (11). Collectively, these data suggest that apoCaM binds to full-length Ca_V_1.2 with a *K*_*D*_ of ∼1 μM, which is outside the physiological concentration range of free CaM (<100 nM) in neurons and cardiomyocytes (15, 16), implying low fractional binding. Furthermore, the recent NMR structure of apoCaM bound to the Ca_V_1.2 IQ-motif revealed an intermolecular salt bridge involving Ca_V_1.2 residue K1662, and the K1662E mutation significantly and selectively weakened apoCaM binding to Ca_V_1.2 (15). At the same time, the K1662E mutation does not affect single-channel Po (15). These previous results suggest that apoCaM may not be the main CaM species to support Ca_V_1.2 activity under basal conditions as proposed previously (12, 13, 17). The current study tested the hypothesis that the Ca_V_1.2 channel may preassociate mostly with a CaM species that is half saturated with Ca^2+^ under basal Ca^2+^ conditions ([Ca^2+^]_i_ = 100 nM).

**Figure 1:**
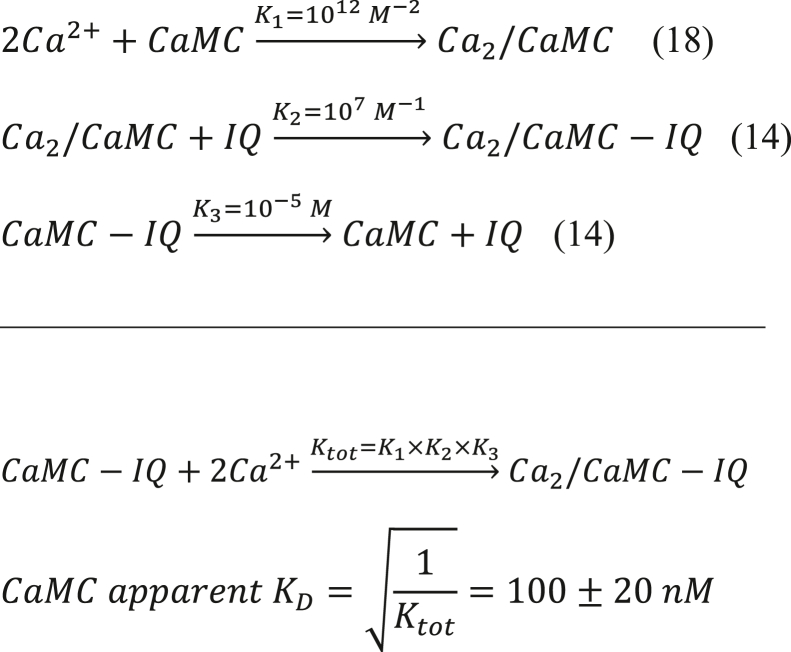
**Apparent Ca**^**2+**^**-binding affinity of the CaM C-lobe bound to IQ (CaMC-IQ).**

**Figure 2:**
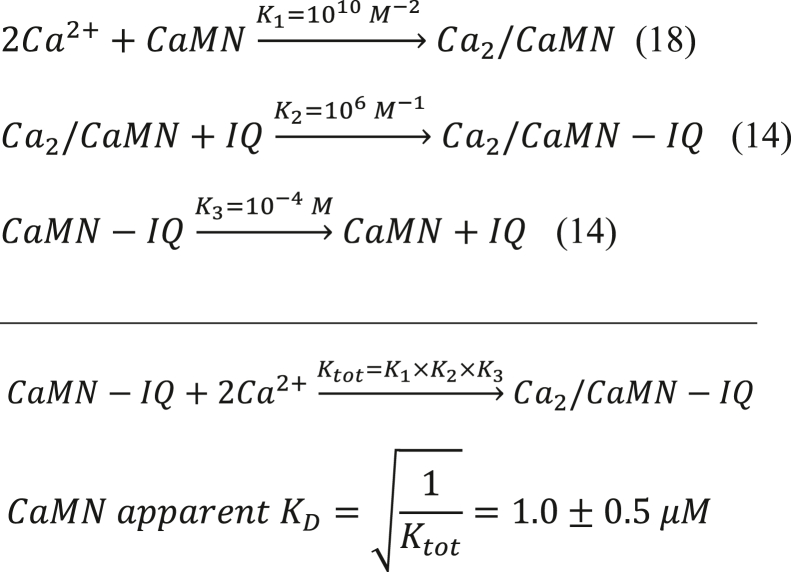
**Apparent Ca**^**2+**^**-binding affinity of the CaM N-lobe bound to IQ (CaMN-IQ).**

**Figure 3:**
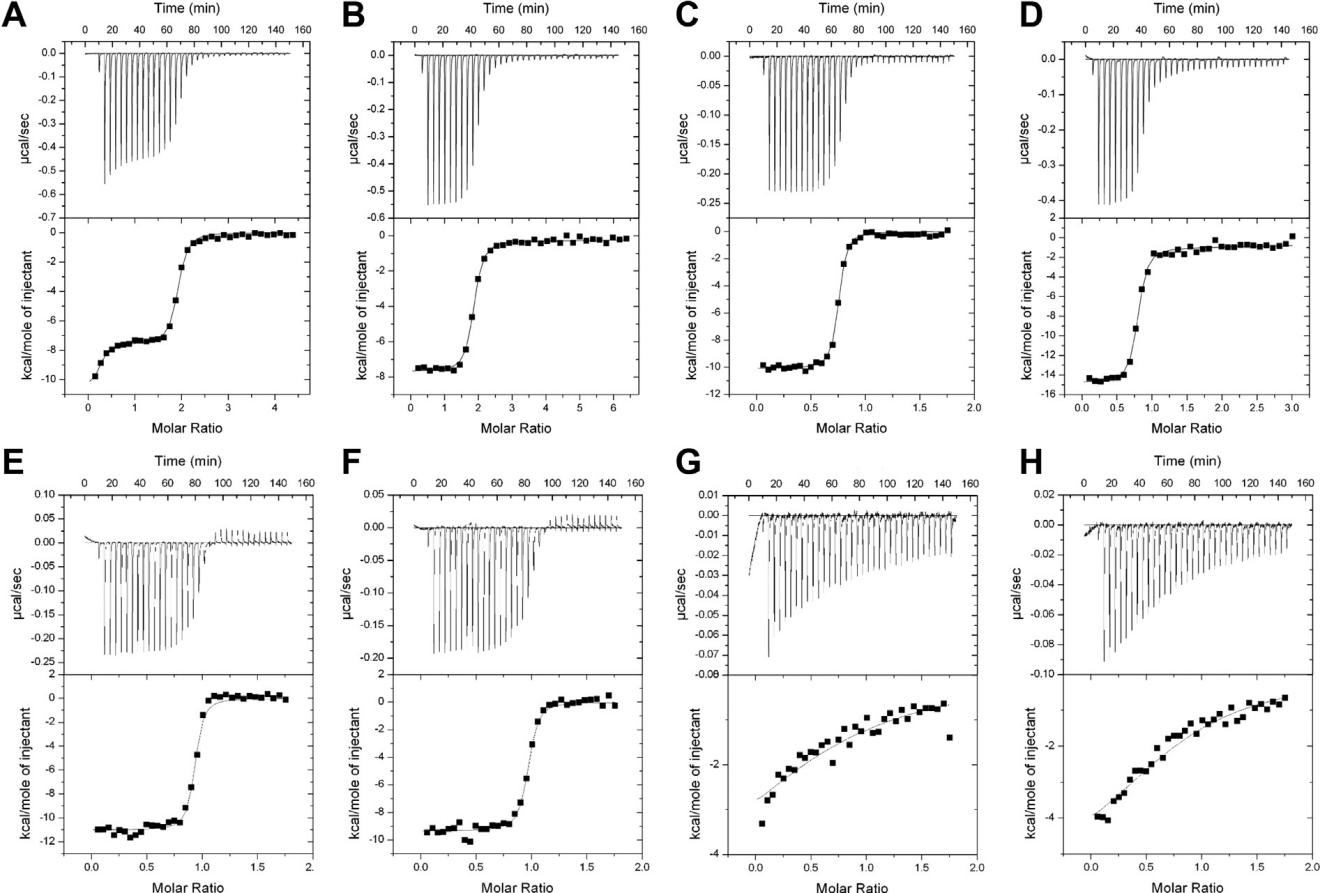
**Isothermal titration calorimetry (ITC) binding assays**. *A* and *B*, ITC measurement of Ca^2+^ binding to CaM_12’_-IQ at 27 °C (*A*) and 37 °C (*B*). The Ca^2+^ binding isotherms at 27 °C and 37 °C were fit to a two-site and one-site model, respectively. The apparent Ca^2+^ affinity ($K_{D}^{app}$) and enthalpy difference (ΔH_1_ and ΔH_2_) are given in Table 1. The CaM_12’_–IQ complex in the sample cell (10 μM at 27 °C or 8.0 μM at 37 °C, 1.5 ml) was titrated with aqueous CaCl_2_ (0.23 mM at 27 °C or 0.30 mM at 37 °C) using 35 injections of 10 μl each. *C*–*D*, ITC measurement of Ca_2_/CaM_12’_ binding to IQ at 27 °C (*C*) and 37 °C (*D*). The dissociation constant (*K*_*D*_) and enthalpy difference (ΔH) for Ca_2_/CaM_12’_ binding to IQ mutants (IQ^WT^, IQ^Y1649A^, IQ^I1654A^, IQ^Y1657D^, IQ^F1658D^, and IQ^F1658A^) are given in Table 3. The binding of Ca_2_/CaM_12’_ to IQ^K1662E^ could not be accurately measured by ITC because IQ^K1662E^ formed aggregated species under the conditions required for ITC. *E*–*H*, ITC measurement at 27 °C of Ca_2_/CaM_12’_ binding to IQ^F1649A^ (*E*), IQ^I1654A^ (*F*), IQ^Y1657D^ (*G*), and IQ^F1658D^ (*H*). The IQ peptide concentrations for WT, Y1649A, and I1654A were each 10 μM (27 °C) or 7.0 μM (37 °C) in 1.5 ml in the sample cell for titration with 0.1 mM Ca_2_/CaM_12’_, and Y1657D and F1658D concentrations were each 50 μM in 1.5 ml for titration with 0.5 mM Ca_2_/CaM_12’_ using 35 injections of 10 μl each. CaM, calmodulin.

In support of our hypothesis, we find that IQ binding to CaM causes a more than 10-fold increase in the apparent Ca^2+^ affinity, which allows Ca^2+^ to bind to the CaM C-lobe under basal conditions (Fig. S1). On the basis of previous binding data (14, 18), the C-lobe under basal conditions is predicted to bind two Ca^2+^ to form a half-calcified state (called Ca_2_/CaM) in which the N-lobe is devoid of Ca^2+^ (19). Indeed, the C-lobe binds Ca^2+^ as well as the IQ motif with 10-fold higher affinity than the N-lobe (14, 18). Using the binding constants from (14, 18) the relative concentrations of apoCaM, CaM intermediate (Ca_2_/CaM), and Ca^2+^-saturated CaM (Ca_4_/CaM) each bound to the IQ as a function of free Ca^2+^ concentration are shown in Fig. S1*A*. The Ca_2_/CaM intermediate species (red trace in Fig. S1*A*) has a significant occupancy of ∼50% at 100 nM Ca^2+^ concentration (basal Ca^2+^ level). Since the apoCaM N-lobe (CaMN) does not bind to IQ under physiological conditions (14), IQ must instead be bound to the C-lobe (CaMC) of Ca_2_/CaM. Using binding constants from (14, 18), we calculate that CaMC-IQ (Fig. 1) and CaMN-IQ (Fig. 2) have apparent *K*_*D*_ values for Ca^2+^ binding of 100 nM and 1.0 μM, respectively:

Thus, the CaM C-lobe is calculated to have a 10-fold higher apparent Ca^2+^ affinity compared to CaM N-lobe. This calculation implies that ∼50% of CaM-IQ complex will have Ca^2+^ bound to its C-lobe under basal conditions ([Ca^2+^]_i_ = 100 nM), whereas the N-lobe should be devoid of Ca^2+^. To test this prediction, we prepared a CaM mutant (D21A/D23A/D25A/E32Q/D57A/D59A/N61A/E68Q, called CaM_12’_) that completely disabled Ca^2+^ binding to EF1 and EF2 but retained normal Ca^2+^ binding to EF3 and EF4. The apparent Ca^2+^ affinity of CaM_12’_ in the presence of saturating IQ peptide under physiological conditions (27 °C and 37 °C) was measured by ITC (Fig. 3, *A* and *B*). The ITC isotherm at 27 °C is biphasic, suggesting possible sample heterogeneity. The major binding component (N_2_ = 1.7 ±0.3 Ca^2+^/protein; Table 1) represents binding of two Ca^2+^ to CaM_12’_-IQ as defined by K_2_, ΔH_2_, and N_2_ (Table 1). The other isotherm component is nonstoichiometric (N_1_ = 0.2 ±0.1 Ca^2+^/protein) and may be an artifact of IQ partial self-association or other sample heterogeneity. Fitting the ITC isotherm with a two-site model reveals a Ca^2+^-binding apparent *K*_*D*_ ($K_{D}^{app}$) of 60 ± 20 nM (Table 1), which agrees within experimental error with the predicted value in Figure 1 and with previously measured values of $K_{D}^{app}$ obtained by UV fluorescence (20). The Ca^2+^-binding ITC isotherm became monophasic at 37 °C, which more accurately demonstrates that two Ca^2+^ bind to CaM_12’_ with a $K_{D}^{app}$ of 72 ± 20 nM and ΔH = -7.7 ± 1 kcal/mol (Fig. 3*B* and Table 1). The relatively high apparent Ca^2+^ affinity $(K_{D}^{app}=72\;nM\;at\;37{}^{\circ}C$) implies that at least 50% of the CaM/IQ complex will have Ca^2+^ bound to EF3 and EF4 ($Y=\frac{\left[Ca^{2+}\right]}{\left[Ca^{2+}\right]+K_{D}}$) at basal Ca^2+^concentrations (∼100 nM). This analysis predicts that slightly more than half of the Ca_V_1.2 channels should be preassociated with the CaM intermediate, Ca_2_/CaM, under basal conditions.

**Table 2:** NMR structural statistics for Ca_2_/CaM_12’_-IQ

NMR restraints	Value (restraint violation)
Short-range NOEs	327 (0.0 ± 0.0)
Long-range NOEs	172 (0.0 ± 0.0)
Hydrogen bonds	81 (not used in water refinement)
Dihedral angles	187 (0.1 ± 0.3)
^1^D_HN_ RDC	24 (0.0 ± 0.0)
RDC Q-factor	0.292
Coordinate precision (Å)^a^	
RMSD backbone atoms	0.83 ± 0.09
RMSD all heavy atoms	1.56 ± 0.1
Deviation from idealized geometry	
Bonds (Å)	0.007 ± 0.000
Angles (°)	0.753 ± 0.012
Impropers (°)	0.927 ± 0.029
Ramachandran Plot (%)	
Favored region	75.0
Allowed region	19.0
Outlier region	7.0
Structure quality^b^	
Clash score	24
Ramachandran outliers	6.6%
Side chain outliers	16.3%

a Coordinate precision was calculated for C-lobe residues 85 to 149.

b Structure quality metrics assessed by MolProbity (51).

### Half-calcified CaM represented by CaM_12’_

The concentration profiles in Fig. S1*A* show that half saturated CaM (Ca_2_/CaM) coexists in an equilibrium mixture with apoCaM and Ca^2+^-saturated CaM (Ca_4_/CaM). At a basal Ca^2+^ concentration of 100 nM, the fractional occupancy of Ca_2_/CaM is calculated to be 55% compared to 7% occupancy of Ca_4_/CaM and 38% occupancy of apoCaM. Therefore, under basal conditions, Ca_2_/CaM cannot be resolved from the other CaM species. To isolate the half Ca^2+^ saturated species, we performed structural studies on the CaM mutant (D21A/D23A/D25A/E32Q/D57A/D59A/N61A/E68Q, called CaM_12’_) that completely disables Ca^2+^ binding to EF1 and EF2 but retains Ca^2+^ binding to EF3 and EF4. The NMR assignments of Ca^2+^-bound CaM_12’_ bound to the IQ peptide (Ca_2_/CaM_12’_-IQ) reveal two downfield NMR peaks assigned to G99 (EF3) and G135 (EF4) that indicate Ca^2+^ is bound to EF3 and EF4 (21). The corresponding Gly residues in EF1 (G26) and EF2 (G62) do not exhibit downfield amide resonances, indicating that EF1 and EF2 in Ca_2_/CaM_12’_-IQ are both devoid of Ca^2+^.

**Figure 4:**
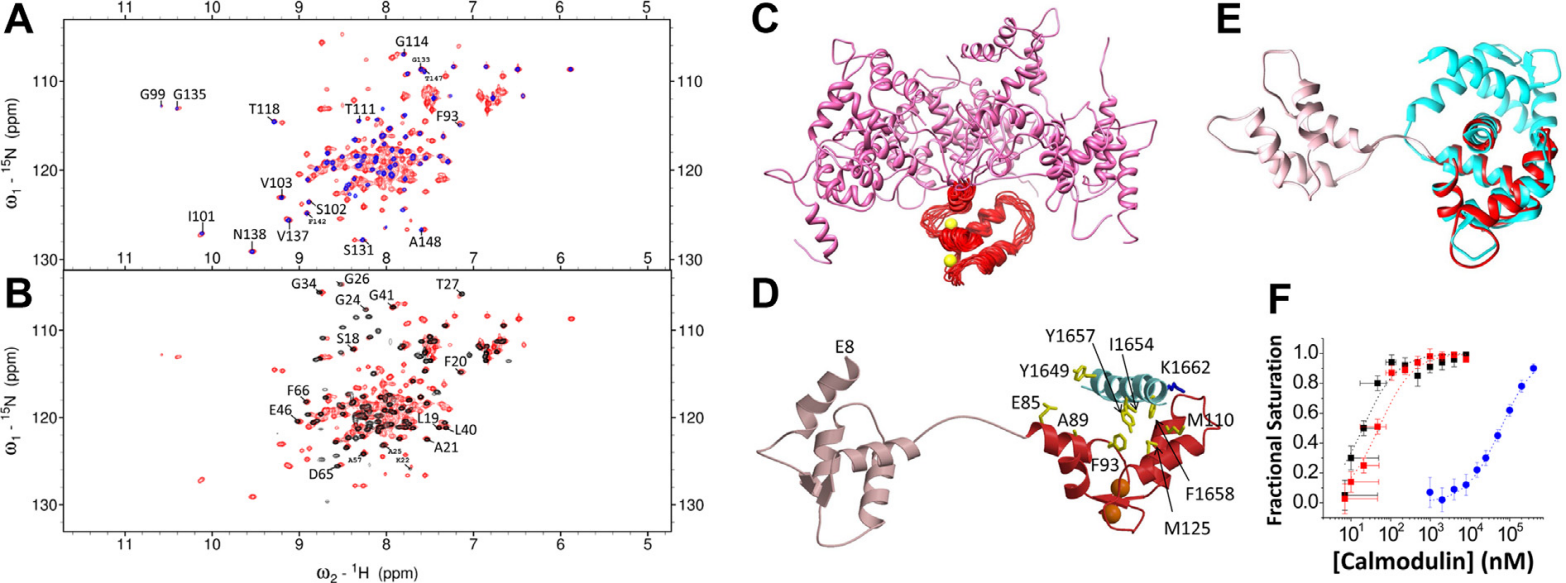
**NMR-derived structures of Ca**_**2**_**/CaM**_**12’**_**-IQ**. *A*, ^15^N-^1^H HSQC NMR spectrum of ^15^N-labeled Ca_2_/CaM_12’_ bound to unlabeled IQ (*red*) is overlaid with the spectrum of Ca^2+^-bound CaM_WT_ C-lobe/IQ complex (*blue*). *B*, NMR spectrum of ^15^N-labeled Ca_2_/CaM_12’_ bound to unlabeled IQ (*black*) is overlaid with the spectrum of Ca^2+^-free CaM_12’_ (*red*). *C*, ensemble of 10 lowest energy NMR structures of Ca_2_/CaM_12’_ (PDB ID: 7L8V). Main chain structures are depicted by a ribbon diagram. Structures of the C-lobe (residues 85–149) are overlaid and highlighted in *red*; N-lobe structures (residues 1–84) are highlighted in *pink*. Bound Ca^2+^ ions are *yellow*. Structural statistics are given in Table 2. *D*, the *lowest energy* structure of Ca_2_/CaM_12’_-IQ complex is shown as a ribbon diagram of Ca_2_/CaM_12’_ bound to the IQ peptide (*cyan*). The CaM N-lobe and C-lobe are highlighted *pink* and *red*, respectively. Side-chain atoms of key residues are depicted by sticks and are colored *yellow* and *blue*. *E*, overlay of the NMR structure of Ca_2_/CaM_12’_-IQ (C-lobe in *red*) with the crystal structure of Ca_4_/CaM (*cyan*, 2BE6). The C-lobe structures overlay with an RMSD of 1.8 Å. *F*, fluorescence polarization assay showing the binding of half Ca^2+^-saturated CaM mutant (Ca_2_/CaM_12’_) with fluorescently labeled IQ peptides (WT: *black*; K1662E: *red*; both: *K*_*D*_ < 100 nM), and of apoCaM binding to Y1657D (*blue*, *K*_*D*_ = 60 μM). CaM, calmodulin; PDB, Protein Data Bank.

The NMR spectrum of Ca_2_/CaM_12’_-IQ is a hybrid of the spectra of Ca^2+^-bound and Ca^2+^-free CaM (Fig. 4, *A* and *B*). The chemical shifts assigned to the CaM_12’_ C-lobe (residues 80–149) of Ca_2_/CaM_12’_-IQ (peaks labeled red in Fig. 4*A*) are nearly identical to those of the isolated Ca^2+^-bound CaM C-lobe bound to IQ (blue peaks in Fig. 4*A*). NMR peaks assigned to CaM_12’_ N-lobe (residues 1–79) of Ca_2_/CaM_12’_-IQ are similar to those of apoCaM_12’_ in the absence of IQ (black peaks in Fig. 4*B*), indicating that the CaM_12’_ N-lobe is Ca^2+^ free and does not interact with the IQ peptide. Thus, only the C-lobe, but not N-lobe, residues in Ca_2_/CaM_12’_ exhibit IQ-induced spectral shifts.

### NMR structure of Ca_2_/CaM_12’_-IQ

NMR spectral assignments for Ca_2_/CaM_12’_-IQ were reported previously (BMRB accession number 27692) (21). These previous NMR assignments were used in the current study to obtain NMR-derived structural restraints from NOESY and residual dipolar coupling (RDC) data (Fig. S4). NMR structures of Ca_2_/CaM_12’_-IQ were then calculated on the basis of distance restraints derived from analysis of NOESY (22) and long-range orientational restraints derived from RDC data (23) as described in the Experimental procedures. The final NMR-derived structures of Ca_2_/CaM_12’_ are overlaid in Figure 4*C* and structural statistics summarized in Table 2. The two domains of Ca_2_/CaM_12’_ (N-lobe in pink and C-lobe in red, Fig. 4*C*) are separately folded and noninteracting, as was seen previously for the NMR structures of apoCaM (24, 25, 26). The overall precision of the NMR ensemble is expressed by a RMSD of 0.83 ± 0.09 Å calculated from the coordinates of the main chain atoms in the C-lobe (Fig. 4*C*) and 0.9 ± 0.1 Å from the main chain atoms in the N-lobe. The lowest energy NMR structure of Ca_2_/CaM_12’_ bound to the IQ peptide is shown in Figure 4*D*. The quality of the NMR structures of Ca_2_/CaM_12’_-IQ was assessed using PROCHECK-NMR (27), which shows that 93% of the residues occur in the allowed or favorable regions from the Ramachandran plot. The NMR structure of the Ca^2+^-bound CaM C-lobe (residues 80–149) of Ca_2_/CaM_12’_-IQ (dark red in Fig. 4, *D* and *E*) looks similar to that observed in the crystal structure of Ca^2+^-saturated CaM bound to the IQ (cyan in Fig. 4*E*) (28). The structure of the Ca^2+^-free CaM N-lobe (residues 1–78) of Ca_2_/CaM_12’_-IQ (light red in Fig. 4*D*) adopts a closed conformation and looks similar to that of apoCaM (26). The IQ peptide was verified by NMR to have a helical conformation (cyan in Fig. 4*D*). In the Ca_2_/CaM_12’_-IQ structure (Fig. 4*D*), the IQ residues (Y1649, I1654, Y1657, and F1658) point toward CaM and make extensive contacts with CaM C-lobe residues (E85, A89, F93, M110, L113, M125). The IQ peptide in the Ca_2_/CaM_12’_-IQ structure does not make any contacts with the Ca^2+^-free N-lobe, in contrast to the crystal structure of Ca_4_/CaM_12’_-IQ (28, 29, 30) where IQ aromatic residues (F1648, Y1649, and F1652) make extensive contacts with N-lobe residues (F13, F69, M73).

**Table 1:** ITC thermodynamic parameters for Ca^2+^ binding to CaM_12’_-IQ

Temp (° C)	N_1_	K_1_ (x10^8^ M^-1^)	ΔH_1_ (kcal/mol)	N_2_	K_2_ (x10^7^ M^-1^)	ΔH_2_ (kcal/mol)	$K_{D}^{app}$ (nM)
27	0.2 ± 0.1	6 ± 4	−10 ± 1	1.7 ± 0.3	1.7 ± 0.4	−7.5 ± 1	60 ± 20
37	−	−	−	1.8 ± 0.3	1.4 ± 0.3	−7.7 ± 1	72 ± 20

### IQ residue K1662 interacts with apoCaM more strongly than Ca_2_/CaM_12’_

The NMR structure of Ca_2_/CaM_12’_-IQ (Fig. 4*D*) looks quite different from the recent NMR structure of apoCaM bound to IQ (15). In the apoCaM-IQ structure, K1662 forms intermolecular salt bridges with CaM residues, E85 and E88. By contrast, K1662 is mostly solvent exposed in the Ca_2_/CaM_12’_-IQ structure and does not contact either E85 or E88 (Fig. 4*D*). This analysis predicts that the Ca_V_1.2 mutation K1662E weakens binding to apoCaM more than it does to Ca_2_/CaM_12’_. Because the K1662E peptide (IQ^K1662E^) was not soluble enough for ITC with Ca_2_/CaM_12’_, we used fluorescence polarization (FP) to measure binding affinity in the nanomolar range. As predicted, titration of the IQ peptides with Ca_2_/CaM_12’_ reached full saturation at 100 nM Ca_2_/CaM_12’_, indicating a *K*_*D*_ < 100 nM for both, IQ^WT^ and IQ^K1662E^ (Fig. 4*F*). It was not possible to more accurately determine the actual *K*_*D*_ because the IQ peptide concentration in Figure 4*F* had to be 100 nM due to limited detection sensitivity. This concentration is much larger than the *K*_*D*_ for IQ^WT^ (16 nM in Table 3) and apparently also for IQ^K1662E^, as binding was clearly saturated at 100 nM for both peptides. The free concentrations of Ca_2_/CaM_12’_ $\left({\left[Ca_{2}/CaM_{12^{\prime }}\right]}_{free}={\left[Ca_{2}/CaM_{12^{\prime }}\right]}_{total}-\left[IQ\right]\times \left(fractional\;saturation\right)\right)$ are within the sample noise level during the first half of the titration when ${\left[Ca_{2}/CaM_{12^{\prime }}\right]}_{free}\;<100\;nM$ (see SD bars in Fig. 4*F*). During the second half of the titration, ${\left[Ca_{2}/CaM_{12^{\prime }}\right]}_{free}$ was above the noise level and the titration curves show clear saturation at 100 nM providing an upper limit of 100 nM for the *K*_*D*_ of both, IQ^WT^ and IQ^K1662E^, consistent with the 16 nM K_D_ for IQ^WT^ as seen by ITC (Table 3). As a result, Ca_2_/CaM_12’_ can bind to IQ^K1662E^ in the nanomolar range in contrast to apoCaM, which binds to IQ^K1662E^ with a *K*_*D*_ in the high micromolar range (60 μM) that is 6-fold higher than that of IQ^WT^ (15). Thus, the K1662E mutation weakens IQ binding to apoCaM to a degree that is outside the physiological range of its concentration (16) (<100 nM), in contrast to the nanomolar binding of IQ^K1662E^ with Ca_2_/CaM_12’_ (Fig. 4*F*). Accordingly, the K1662E mutation can be used to selectively disable apoCaM binding to Ca_V_1.2, while retaining Ca_V_1.2 binding to Ca_2_/CaM.

**Table 3:** Dissociation constants (*K*_*D*_), enthalpy differences (ΔH), and stoichiometry (n) for Ca_2_/CaM_12’_ binding to IQ variants as measured by ITC

Temp (° C)	IQ peptide	*K*_*D*_ (nM)	ΔH (kcal/mol)	n-value
37	WT	37 ± 10	−15 ± 0.2	0.76 ± 0.25
27	WT	16 ± 5	−10 ± 0.2	0.77 ± 0.25
27	Y1649A	26 ± 5	−9.7 ± 0.2	0.88 ± 0.25
27	I1654A	60 ± 10	−9.2 ± 0.2	0.89 ± 0.25
27	Y1657D	8000 ± 900	−5.6 ± 0.7	0.72 ± 0.5
27	F1658A	32 ± 5	−9.5 ± 0.2	1.0 ± 0.25
27	F1658D	4000 ± 700	−5.9 ± 0.7	0.8 ± 0.5

The errors are the SD calculated from three independent trials.

### IQ residues Y1649, I1654, Y1657, and F1658 interact with Ca_2_/CaM_12’_

The NMR structure of Ca_2_/CaM_12’_-IQ reveals intermolecular contacts with IQ residues, Y1649, I1654, Y1657, and F1658, that are each located on the same side of the IQ helix pointing toward the Ca^2+^-occupied C-lobe of Ca_2_/CaM_12’_ (Fig. 4*D*). As predicted by this analysis, the IQ peptide mutants IQ^Y1649A^, IQ^F1654A^, IQ^Y1657D^, and IQ^F1658D^ each exhibited weaker binding to Ca_2_/CaM_12’_ compared to IQ^WT^. The *K*_*D*_ was 16 ± 5 nM for IQ^WT^, 26 ± 5 nM for IQ^Y1649A^, 60 ± 10 nM for IQ^I1654A^, 8000 ± 10 nM for IQ^Y1657D^, 4000 ± 10 nM for IQ^F1658D^, and 32 ± 5 nM for IQ^F1658A^ (Fig. 3, *C* and *E*–*H* and Table 3). These findings validate our structural analysis and verify that Y1657 makes the strongest contact with CaM.

The highly exothermic binding of the IQ peptide to Ca_2_/CaM_12’_ (ΔH° = -15 kcal/mol in Figure 3*D* and Table 3) predicts the *K*_*D*_ to increase by 2.3-fold when the temperature is increased from 27 °C to 37 °C. As predicted, the *K*_*D*_ for IQ binding to Ca_2_/CaM_12’_ increased from 16 ± 5 nM (at 27 °C) to 37 ± 10 nM at 37 °C. Also, the temperature dependence of ΔH (-10 kcal/mol at 27 °C *versus* −15 kcal/mol at 37 °C) indicates a negative ΔC_p_ value, which is consistent with the relatively large change in solvent accessible hydrophobic surface area that occurs when Ca_2_/CaM_12’_ binds to the IQ peptide.

### The K1662E mutation affects binding of apoCaM but not CDI of Ca_V_1.2

**Figure 5:**
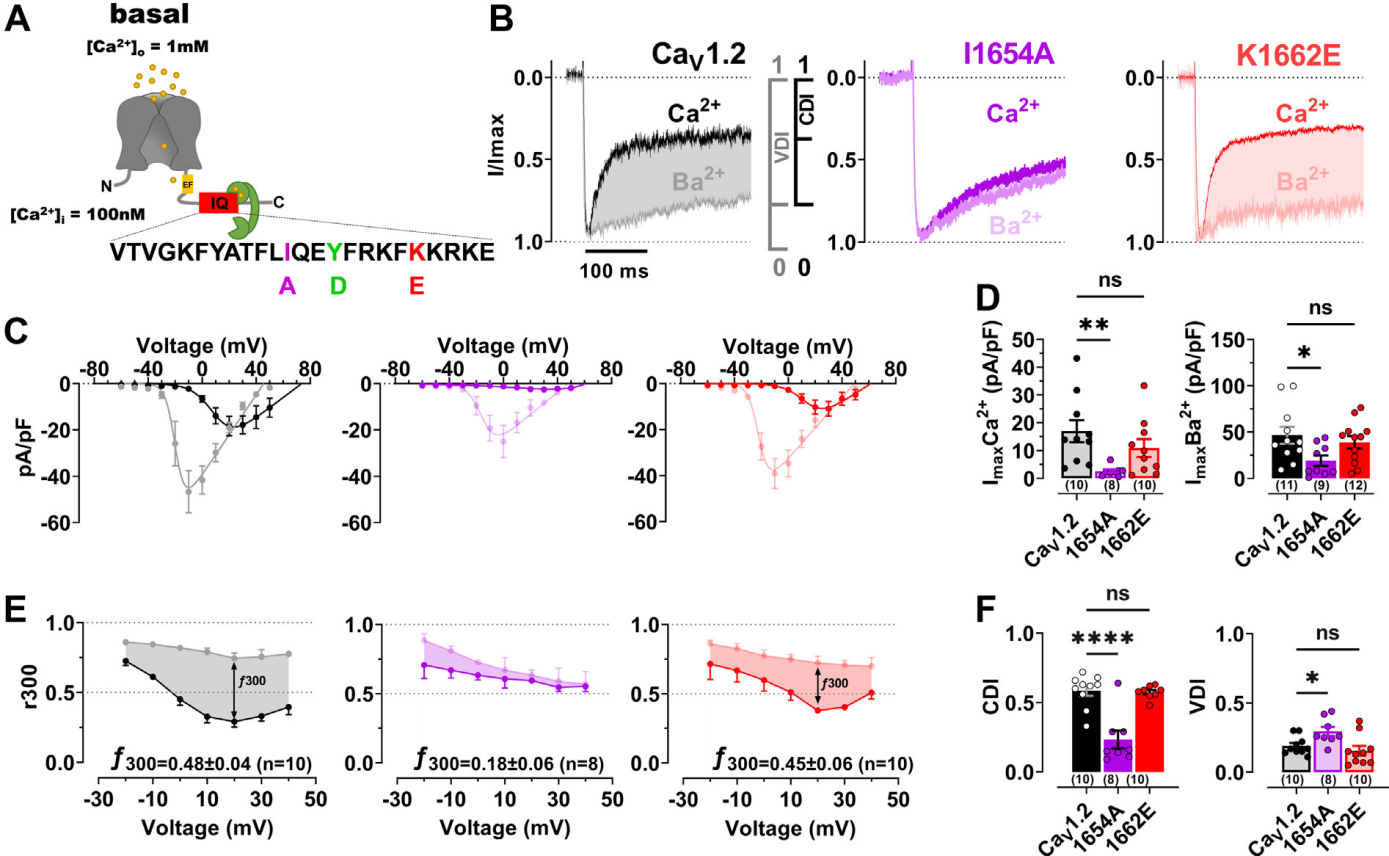
**Effects of IQ mutants I1654A and K1662E on Ca**_**V**_**1.2 activity and inactivation**. *A*, topology of the hypothetical Ca_V_1.2 Ca^2+^ channel pore and localization of the IQ domain and its mutations in the α_1_1.2 subunit. At rest with [Ca^2+^]_i_ ≤100 nM the C-lobe (*green*) of half-calcified Ca^2+^/CaM is predicted to bind to the C-terminal portion of the IQ motif, making hydrophobic contacts with I1654 and Y1657 but not with K1662. *B*–*F*,) HEK 293T/17 cells were transfected with α_1_1.2, α_2_δ1, and β_2A_. Shown are representative whole-cell current traces (*B*), population data of current-voltage relationships (I/V curves) (*C*), their respective peak current density plots (*D*), and currents remaining after 300 ms of depolarization (r300; *bottom*) of I_Ba_ (10 mM Ba^2+^; *gray* or *light colors*) and I_Ca_ (10 mM Ca^2+^; *black* or dark colors), for WT (*black*), I1654A (*purple*), and K1662E (*red*). Statistical significance was determined by a one-way ANOVA with Bonferroni correction, (∗*p* < 0.05, F(DFn, DFd), F(2,29), F3.3and *p*∗∗<0.01, F(DFn, DFd), F(2,25) = 4.9. *E*, peak currents in (*B*) were normalized to the respective current maxima (Imax). Shaded areas indicate differences between I_Ba_ and I_Ca_ as read out for CDI (f300: difference between I_Ba_ and I_Ca_ remaining after 300 ms). Quantification of peak current densities of I_Ba_ and I_Ca_ at potential of respective Imax reveals a strong decrease in current density for I1654A but not K1662E *versus* WT (*D*). *F*, quantification of CDI and VDI reveals a strong decrease in CDI for I1654A but not K1662E *versus* WT. Additionally, I1654A showed a robust and significant increase in VDI *versus* WT, whereas K1662E remained unaffected. Numbers in parenthesis under bars reflect n independent recordings and error bars SEM (∗*p* < 0.05, F(DFn, DFd), F(2,25) = 5.5, and ∗∗∗∗*p* < 0.0001, F(DFn, DFd), F(2,23) = 20.9, One-way ANOVA with Bonferroni correction). CaM, calmodulin; CDI, Ca2+ dependant inactivation; VDI, voltage dependent inactivation.

The aforementioned analysis suggests that K1662E retains binding to Ca_2_/CaM_12’_ but not apoCaM under physiological conditions (*i.e*., with free CaM < 100 nM (16)) (Fig. 4*F*). This differential effect informs interpretation of recently published data that showed that the K1662E mutation has no effect on Po (15), while the I1654A mutation, which affects binding of both apoCaM and Ca/CaM, decreased Po by 6-fold (15). A similar effect has been seen for an analogous Ile to Ala mutation in the closely related Ca_V_1.3 (17). Collectively, these findings suggest that CaM promotes Po when it forms a complex with Ca_V_1.2 with Ca^2+^ bound to EF3 and EF4 to give rise to a half-saturated Ca_2_/CaM state in this complex. To further test the idea of preassociation of half Ca^2+^-saturated Ca_2_/CaM with Ca_V_1.2 at basal Ca^2+^ concentrations, we wanted to compare CDI of Ca_V_1.2^K1662E^ with WT and also Ca_V_1.2^I1654A^, which served as a well-established reference point for loss of CDI (8, 13, 17). For that purpose, we measured whole-cell current density for I_Ba_ and I_Ca_. Consistent with the earlier Po analysis, I_Ba_ and I_Ca_ were reduced by the I1654A but not K1662E mutation (Fig. 5, *A*–*D* and Table S1*A*). Strikingly, the K1662E mutation had no significant effect on CDI (nor on voltage-dependent inactivation), in contrast to the I1654A mutation, which reduced CDI by ∼75% (Fig. 5, *B*, *E*, and *F* and Table S1*B*). The small, remaining CDI seen for the I1654A mutant channel may be due to N-lobe effects such as its binding to the N terminus of the Ca_V_1.2 α_1_ subunit (31). The differential effect on I_Ba_, I_Ca_, and CDI by the K1662E *versus* I1654A mutation is consistent with the differential effect of the K1662E *versus* I1654A mutation on Po (15) and suggests that formation of a complex of Ca_V_1.2 with half Ca^2+^-saturated Ca_2_/CaM is important for Po and for predisposing Ca_V_1.2 to CDI.

### The Y1657D mutation strongly affects binding of half-saturated Ca_2_/CaM as well as I_Ba_, I_Ca_, Po, and CDI of Ca_V_1.2

**Figure 6:**
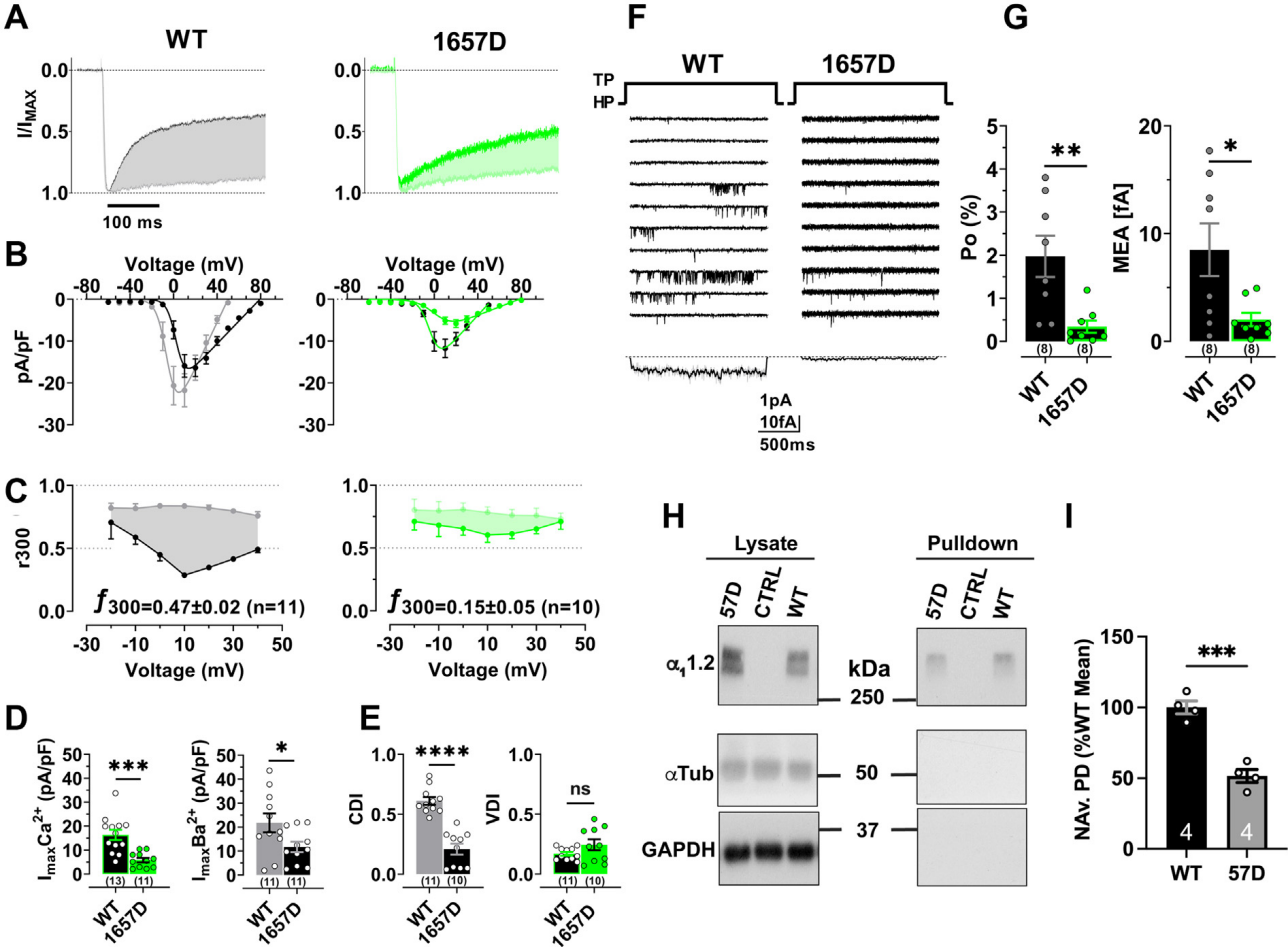
**Effects of IQ mutant Y1657D on Ca**_**V**_**1.2 activity, inactivation, and surface expression.** HEK 293T/17 cells were transfected with α_1_1.2, α_2_δ1, and β_2A_. Shown are representative whole-cell current traces (*A*), population data of I/V curves (*B*) currents remaining after 300 ms of depolarization (r300; *bottom*) of I_Ba_ (*gray* or *light green*), and I_Ca_ (*black* or *dark green*), for WT (*black*) and Y1657D (*green*) (*C*), and peak current density plots (*D*). Peak currents in (*A*) were normalized to the respective current maxima (Imax). Shaded areas indicate differences between I_Ba_ and I_Ca_ as read out for CDI (f300: difference between I_Ba_ and I_Ca_ remaining after 300 ms). *D*, quantification of peak I_Ba_ and I_Ca_ at potential of respective Imax reveals a strong decrease in current density for Y1657D *versus* WT. *E*, quantification of CDI and VDI reveals a strong decrease in CDI but not VDI for Y1657D *versus* WT. *F*, 10 consecutive representative single-channel traces of WT and Y1657D. Below: mean ensemble average currents (MEA) calculated from a total of 857 superimposed traces for WT (n = 8 cells) and 1366 traces for Y1657D (n= 8 cells). *G*, quantification of single-channel open probability Po (*left*) and MEA (*right*) reveals a strong decrease in channel activity for Y1657D *versus* WT. Statistical difference was determined by an unpaired, two-tailed Student’s *t* test, *p*∗<0.05, *p*∗∗<0.01, *p*∗∗∗<0.001 and *p*∗∗∗∗<0.0001. *H*, surface biotinylation of Ca_V_1.2 was followed by Neutravidin pull downs and immunoblotting (*right panels*) with antibodies against the proteins indicated at the left. Left panels show immunoblots of total lysate. Tubulin (α-Tub) and GAPDH were used as loading controls for lysate samples (*left*) and assessment of membrane integrity (*right*; all *left* and *right panels* were from same gels and exposures). Absence of tubulin and GAPDH immunoreactivity indicates that the biotin reagent did not leak into cells ruling out biotinylation of intracellular proteins. *I*, quantification of α_1_1.2 immunosignals in Neutravidin pull downs (NAv.PD) normalized to WT α_1_1.2 (set to 100%). The 1657D α_1_1.2 mutant exhibits a decrease in surface biotinylation relative to the WT subunit (n = 4; *p* = 0.0003, two-tailed unpaired *t* test). Numbers in parenthesis under bars or inside bars reflect “n” independent recordings or pull downs and error bars SEM (∗*p* < 0.05, ∗∗*p* < 0.01, ∗∗∗*p* < 0.001, ∗∗∗∗*p* < 0.0001, *t* test). VDI, voltage dependent inactivation.

Our new Ca_2_/CaM_12’_-IQ structure indicates that Y1657 makes the most and closest contacts among all IQ residues with Ca_2_/CaM_12’_ (Fig. 4). In support of its central role in mediating this interaction, binding studies indicate that the Y1657D mutation has the strongest negative effect on the affinity of the Ca_2_/CaM_12’_-IQ interaction of all tested IQ peptides (*K*_*D*_ for IQ^WT^ is 16 nM and for IQ^Y1657D^ 8 μM; Table 3). The Y1657D mutation decreased whole-cell currents, I_Ba_ and I_Ca_, as well as CDI with no apparent effect on voltage dependent inactivation (Fig. 6, *A*–*E*). Single-channel recordings show a remarkably strong decrease in Po for Y1657D *versus* WT Ca_V_1.2 (Fig. 6, *F* and *G*). This loss in Po and CDI is comparable to similarly strong effects for the I1654A mutation on Po (15) and CDI (9) but the K1662E mutation, which specifically affects apoCaM but not Ca/CaM binding, did not affect Po (15) or CDI (Fig. 5). The decrease in Po is also well reflected when calculating the ensemble averages of unitary single-channel currents (Fig. 6*F* and Table S2). To test whether there is also a change in channel surface expression in addition to a decrease in Po of individual channels, we conducted surface biotinylation experiments. We determined that Ca_V_1.2 surface expression was reduced by almost 50% (Fig. 6, *H* and *I*), which can explain some, but not all, of the 80% loss in Po.

### CaM intermediate (Ca_2_/CaM) increases Po of Ca_V_1.2

To further analyze the role of CaM in Po, we ectopically expressed CaM in HEK 293T cells. Although this approach has been used before to define the role of CaM in CDI, the level to which exogenous CaM was expressed in these CDI studies had not been thoroughly assessed (32). Thus, we investigated whether the expression of CaM_34_ (described by (8)) was sufficient to allow detection of an effect (*i.e*., many fold greater than endogenous CaM) by immunoblotting extracts of 293T cells transfected with Ca_V_1.2 expression constructs ± WT CaM or CaM_34_ plasmids (Fig. S2). We found that overexpression of WT compared to endogenous CaM is about ∼10 fold, while CaM_34_ is ∼20 fold (Fig. S2, *A*–*D*). To test whether ectopic expression of CaM affects levels of endogenous CaM, we expressed YFP-tagged WT CaM or CaM_34_, which migrate at an M_R_ of ∼ 45 kDa (verified by anti-YFP immunoblotting; Fig. S2*E*). Probing immunoblots with anti-CaM identifies a prominent 45 kDa band and a weaker signal for the endogenous 17 kDa band. Comparison of the 17 kDa band in mock-transfected (no CaM vectors) cell lysate to the same M_R_ immunoreactive band in the CaM plasmid-transfected samples did not indicate a significant effect of ectopic CaM on endogenous CaM levels (Fig. S2, *E* and *F*).

**Figure 7:**
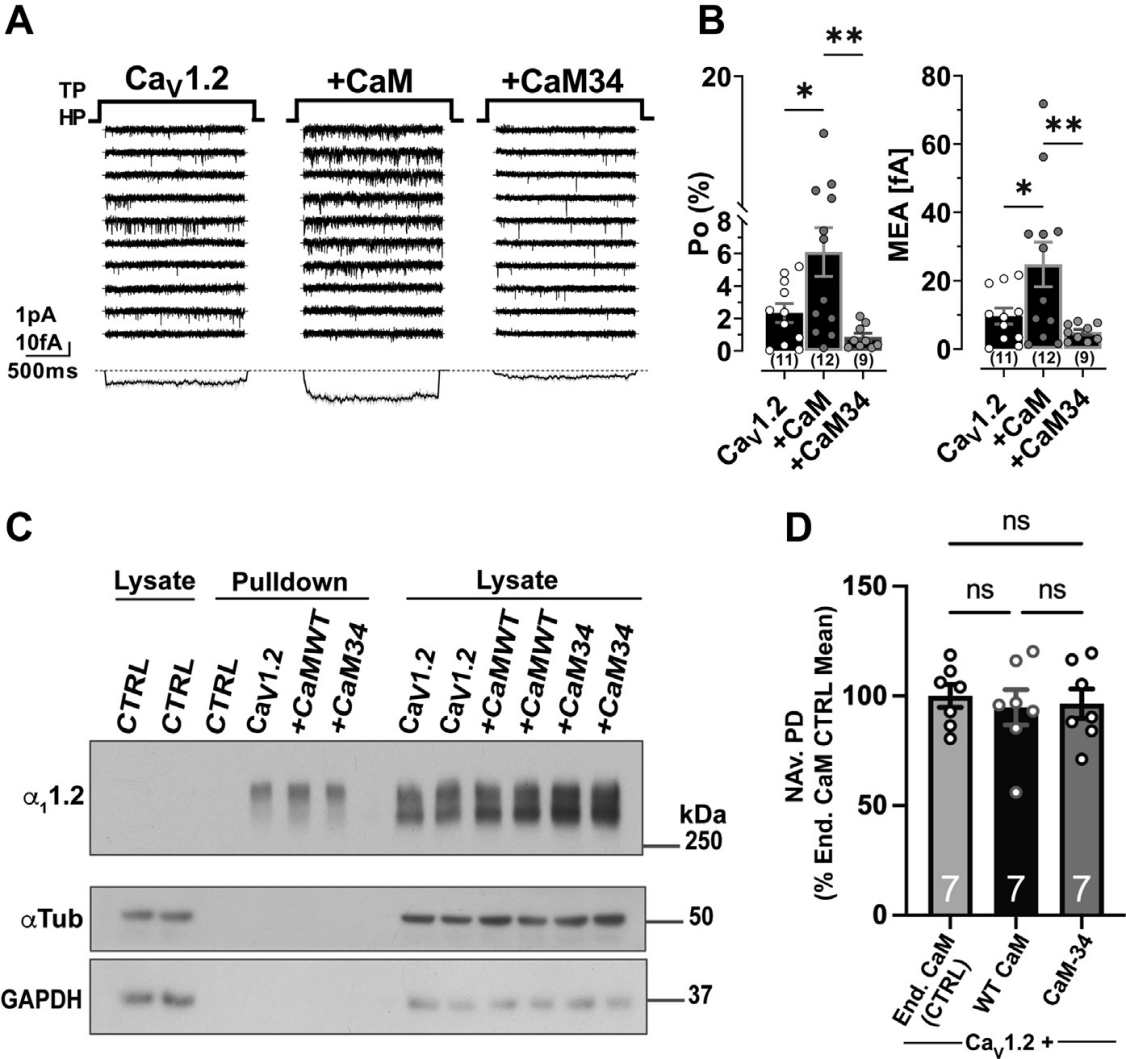
**Effects of ectopic expression of WT CaM and CaM**_**34**_**on Ca**_**V**_**1.2 activity, inactivation, and surface expression.** HEK 293T/17 cells were transfected with α_1_1.2, α_2_δ1, and β_2A_ plus, if indicated, WT CaM or CaM_34_. *A*, 10 consecutive representative single-channel traces of WT Ca_V_1.2 expressed alone (*left*) or together with WT CaM (*middle*) or CaM_34_ (*right*). *Bottom*: MEA calculated from a total of 2009 superimposed traces for Ca_V_1.2 expressed without CaM (n = 11 cells), 2327 traces for Ca_V_1.2 expressed with WT CaM (n = 12 cells), and 1655 traces for Ca_V_1.2 expressed with CaM_34_ (n = 9 cells). *B*, quantification of Po (*left*) and MEA (*right*) reveals a strong increase in channel activity for ectopic expression of WT CaM but not CaM_34_ (numbers in parenthesis under bars reflect n independent recordings and error bars SEM; ∗*p* < 0.05, and ∗∗*p* < 0.01, F(DFn, DFd), F(2,29) = 6.8 and ∗*p* < 0.05 and ∗∗*p* < 0.01, F(DFn, DFd), F(2,29) = 5.4, one-way ANOVA with Bonferroni correction). *C*, surface biotinylation of Ca_V_1.2 was followed by Neutravidin pull downs (*middle of blot*) and immunoblotting with antibodies against the proteins indicated at the *left*. *Right side* shows respective total lysate samples in duplicate and left side total lysate samples from mock-transfected cells. Cells expressing Ca_V_1.2 only or CFP-tag empty vector only were used as controls (CTRL). Tubulin (α-Tub) and GAPDH were used as loading controls for lysate samples and assessment of membrane integrity for pull-down samples. Absence of tubulin and GAPDH immunoreactivity ruled out biotinylation of intracellular proteins. *D*, quantification of α_1_1.2 immunosignals in Neutravidin pull downs (NAv.PD) normalized to mean (set to 100%) of the signal in Ca_V_1.2 only samples (control, only endogenous CaM); n = 7; one-way ANOVA (F = 0.1547, *p* = 0.8578), followed by Tukey’s post-hoc test, ns = *p* > 0.05). CaM, calmodulin; MEA, mean ensemble average.

Consistent with earlier work on Ca_V_1.3 by Adams *et al*. (17), we find that overexpression of WT CaM strongly increases Po by ∼300% as compared to expression of Ca_V_1.2 alone (Fig. 7, *A* and *B* and Table S3). This effect could be due to increased binding of apoCaM, half Ca^2+^-saturated Ca_2_/CaM, or both. Because earlier work did not differentiate between these possibilities (17), we tested the effect of ectopic expression of CaM_34_ and found no increase at all in Po as compared to expression of Ca_V_1.2 alone. This result demonstrates that Ca^2+^ binding to EF3 and EF4 in CaM is essential for promoting the increased Po. There was no detectable effect on surface expression of Ca_V_1.2 by either WT CaM or CaM_34_ (Fig. 7, *C* and *D* and Table S3). Given the ∼20-fold higher expression levels of CaM_34_ *versus* endogenous CaM, it seems especially remarkable that this overexpression had no effect at all on Po when a lesser degree of overexpression of WT CaM induced a ∼3-fold increase in Po (Fig. 7). Collectively, these data indicate that binding of Ca_2_/CaM and not apoCaM to Ca_V_1.2 at basal Ca^2+^ concentrations mediates the observed increase in Po.

## Discussion

Preassociation of CaM with Ca_V_1.2 and the highly homologous Ca_V_1.3 under basal conditions has been suggested to both augment channel activity at low Ca^2+^ levels (17) and facilitate rapid CDI (8, 9). We provide multiple lines of evidence that Ca_V_1.2 preassociates with half-calcified Ca_2_/CaM that contains two Ca^2+^ bound to the CaM C-lobe. The fact that the CaM_34_ mutant abolished the 300% increase in channel open probability of Ca_V_1.2 caused by WT CaM (Fig. 7, *A* and *B*) implies that Ca^2+^ binding to EF3 and EF4 (hence half-calcified CaM) is essential for Ca_V_1.2 channel function. Also, our binding analysis reveals that IQ binding to CaM increases the apparent Ca^2+^ affinity by at least 10-fold (see Fig. 1 and Table 3), consistent with observations from previous binding studies (14, 20). Hence, the IQ-bound CaM C-lobe is more than 50% saturated with Ca^2+^ at basal Ca^2+^ concentrations when CaM is saturated with the IQ peptide (Fig. S1). The concentration of free endogenous CaM inside a cell is estimated to be between 50 to 100 nM (16). As Ca_2_/CaM binds to the IQ motif with a *K*_*D*_ of 16 nM, we estimate that ∼50% of Ca_V_1.2 is bound to Ca_2_/CaM under basal conditions, which would put the channel regulation by CaM in the middle of its dynamic range.

The NMR structure of Ca_2_/CaM_12’_-IQ reveals that half Ca^2+^-saturated CaM (Ca_2_/CaM) has a closed conformation (26) in the Ca^2+^-free N-lobe and a Ca^2+^-bound open conformation (28) in the C-lobe (Fig. 4). The N- and C-lobe structures of Ca_2_/CaM_12’_-IQ are separately folded and do not exhibit interdomain contacts (Fig. 4*C*). The two separate lobes in Ca_2_/CaM_12’_-IQ are dynamically independent, similar to apoCaM (26, 33, 34). The Ca^2+^-free N-lobe structure in Ca_2_/CaM_12’_-IQ does not interact with the IQ peptide, in contrast to the IQ contacts with the N-lobe observed in the crystal structure of Ca^2+^-saturated CaM (28, 29, 30). The IQ peptide binds exclusively to the Ca^2+^-bound C-lobe of Ca_2_/CaM (Fig. 4*D*), whose structure is similar to the C-lobe of Ca_4_/CaM bound to the IQ (Fig. 4*E*) (28, 29, 30). The IQ peptide bound to Ca_2_/CaM_12’_ is rotated 180° compared to the orientation of the IQ bound to apoCaM (15). The opposite binding orientation may explain in part why the IQ binds to Ca_2_/CaM with at least 100-fold higher affinity (Fig. 4*F*) compared to that of apoCaM (14, 15). The contrasting binding orientation also suggests why the preassociation of Ca_V_1.2 with Ca_2_/CaM (rather than with apoCaM) predisposes Ca_V_1.2 for CDI. Since Ca_2_/CaM and Ca_4_/CaM both bind to Ca_V_1.2 with the same orientation, CaM can remain bound to Ca_V_1.2 upon Ca^2+^ influx to facilitate rapid CDI. By contrast, preassociated apoCaM would first need to dissociate from Ca_V_1.2 upon Ca^2+^ influx and then subsequently rebind in the conformation adopted by Ca^2+^-saturated Ca_4_/CaM to engage CDI (28, 29, 30). This unbinding of apoCaM and rebinding of Ca_4_/CaM would likely prevent rapid CDI and defeat the purpose of the CaM preassociation.

Our functional analysis fully supports the relevance of prebinding of Ca_2_/CaM to the Ca_V_1.2 IQ motif. The K1662E mutation, which impaired binding of apoCaM (15) but retained binding to Ca_2_/CaM at physiological CaM concentrations of ∼100 nM (16) (Fig. 4*F*), did not affect Po (15), CDI, I_Ba_, or I_Ca_ (Fig. 5). Furthermore, the Y1657D mutation impaired binding of apoCaM (*K*_*D*_ = 60 μM, Fig. 4*F*), as well as Ca_2_/CaM (*K*_*D*_ = 8 μM, Table 3), and reduced Po, CDI, I_Ba_, and I_Ca_ (Fig. 6). We also tested the effect of ectopic expression of CaM_34_ and CaM_1234_. Consistent with the earlier work on the closely related Ca_V_1.3 (17), overexpression of WT CaM strongly augmented Po (Fig. 7 and Table S3). The main finding of these authors (17) was that substitution of the eponymous Ile in the IQ motif by Met reduced Po and overexpression of WT CaM rescued this loss. Because mutating this Ile reduces binding of apoCaM, these authors concluded that it is apoCaM that binds to the IQ motif under resting Ca^2+^ concentrations to augment Po. However, they did not test the effect of overexpression of CaM_1234_ or CaM_34_ on single channel activity as is required for measuring Po and thus did not rule out that Po is driven by the binding of Ca_2_/CaM, whose binding to the IQ motif is also strongly impaired by mutating this Ile. Importantly, we found that neither CaM_34_ (Fig. 7 and Table S3) nor CaM_1234_ (Fig. S3 and Table S4) increased Po, despite the fact that the exogenous CaM levels were much higher (by 20-fold) than that of endogenous CaM. In addition, the differential effects of (1) the K1662E mutation on Ca_V_1.2 binding to apoCaM *versus* Ca_2_/CaM; (2) K1662E *versus* Y1657D on Po and CDI; and (3) WT CaM *versus* CaM_34_ or CaM_1234_ on Po collectively indicate that preassociated Ca_2_/CaM is an important factor in determining channel Po.

As discussed previously, we estimate that ∼50% of Ca_V_1.2 is occupied by Ca_2_/CaM with little occupancy by apoCaM due to its low concentration in the cytosol (50–100 nM (16)) and low affinity binding to the IQ (*K*_*D*_ = 10 μM (15)) and full-length Ca_V_1.2 (*K*_*D*_ = 1 μM (11)). How then can the remainder of the Ca_V_1.2 population possess a reasonable level of activity? We previously found that binding of α-actinin to the IQ motif also strongly augments Po (15). Thus, we propose a model in which Ca_V_1.2 is either occupied by α-actinin, which at the same time anchors Ca_V_1.2 at the cell surface and especially in dendritic spines where α-actinin is concentrated (35) or by Ca_2_/CaM. Accordingly, in addition to strongly promoting Po, α-actinin also augments the Ca_V_1.2 surface expression (15), perhaps by connecting to F-actin (36). On the other hand, Ca_2_/CaM augments Po with apparently little if any effect on surface expression. Channel occupancy by Ca_2_/CaM could be increased upon modest increases of basal Ca^2+^ influx potentially in a positive feedback loop at low Ca^2+^ levels and low channel activity. However, prolonged displacement of α-actinin by Ca_4_/CaM also triggers endocytosis of Ca_V_1.2 as a negative feedback mechanism (35). At this point, we cannot be certain about how α-actinin and CaM intersect at the IQ motif to govern Ca_V_1.2 activity, and much needs to be learned with respect to the exact function of these interactions.

In conclusion, our analysis provides novel mechanistic insight into preassociation of CaM with Ca_V_1.2 and its role in controlling channel activity and CDI. These findings are not only of functional relevance for understanding the physiological effects of Ca_V_1.2 but also inform the current understanding of pathological events such as arrhythmias due to impaired CDI (37, 38).

## Experimental procedures

### CaM_12’_ mutagenesis and purification and IQ peptide for NMR

The CaM_12’_ mutation ((D21A/D23A/D25A/E32Q/D57A/D59A/N61A/E68Q) was introduced into *Xenopus* CaM complementary DNA by PCR QuickChange procedure (39). The mutated complementary DNA was inserted into the NcoI/BamHI sites of a pET11d vector and verified by automated Sanger sequencing. The recombinant CaM_12’_ protein was expressed from a pET11d vector in a BL21(DE3) Codon Plus *Escherichia coli* strain (Stratagene) and purified as described previously (40). The Ca_V_1.2 IQ peptide (residues 1644–1664) was purchased from ChinaPeptides. The peptide was dissolved in d_6_-dimethyl sulfoxide to give a peptide concentration of 7.8 mM. The peptide concentration was determined by measuring absorbance at 280 nm with ε_280_ = 2980 M^−1^ cm^−1^. An aliquot of peptide (1.5 equivalents) was added to a dilute solution of CaM_12’_ (50 μM protein dissolved in 20 mM 2-amino−2−hydroxymethyl-propane-1,3-diol-d11 (Tris-d_11_) with 95% H_2_O/5% D_2_O). The complex was then concentrated to a final concentration of 500 μM in a final volume of 500 μl for NMR experiments. The 1.5-fold excess of IQ peptide in the NMR sample of Ca_2_/CaM_12’_-IQ was necessary to minimize the occupancy of a 2:1 complex, in which two molecules of CaM_12’_ were bound to one IQ. The HSQC spectrum of a sample that contained an equal concentration of CaM_12’_ and IQ revealed two distinct peaks for each C-lobe residue of CaM_12’_ (Fig. S4*D*). The most intense peak represented a 1:1 complex (∼90% occupancy) and a weaker second peak (marked by arrows in Fig. S4*D*) represented a second CaM_12’_ molecule bound to IQ in a 2:1 complex (∼10% occupancy). The relative occupancy of the 2:1 complex could approach nearly 100% when the CaM_12_ concentration is more than 10-fold higher than that of Ca_V_1.2, like what exists inside HEK293 cells used in the Ca_V_1.2 electrophysiological experiments (Fig. S2). The 2:1 complex likely consists of a single IQ peptide that binds tightly to a Ca^2+^-bound C-lobe on one side of the IQ helix (CaM_12’_ C-lobe contacting I1654 and Y1657) as well as a second CaM_12’_ C-lobe that binds with lower affinity to the opposite side of the IQ helix (CaM_12’_ C-lobe contacting F1648 and F1652). The binding of a second C-lobe from CaM_12’_ mimics the binding of the Ca^2+^-bound N-lobe from WT CaM. Therefore, we suggest that the CDI observed for Ca_V_1.2 in the presence of CaM_12’_ (13) is likely an artifact of the formation of a 2:1 complex in HEK293 cells involving two of the overexpressed CaM_12’_ molecules bound to a single Ca_V_1.2.

### ITC

ITC experiments were performed using a VP-ITC calorimeter (Micro-Cal) at 27 °C and 37 °C. The data were acquired and processed with MicroCal software (https://www.originlab.com) as described previously (41). The first data point from each ITC isotherm was deleted because the amount of injectant delivered during the first injection has significant error caused by a dead volume void in the injection syringe. For ITC experiments in Figure 3, *A* and *B*, samples of Ca^2+^ (injectant) and CaM_12’_–IQ complex (titrant) were prepared by exchanging each into buffer containing 20 mM Tris, pH 7.4, and 100 mM KCl. The CaM_12’_–IQ complex in the sample cell (10 μM at 27 °C or 8.0 μM at 37 °C in 1.5 ml) was titrated with aqueous CaCl_2_ (0.23 mM at 27 °C or 0.3 mM at 37 °C) using 35 injections of 10 μl each. For the ITC experiments in Fig. 3, *C*, *E*–*H*, samples of Ca_2_/CaM_12’_ (injectant) and IQ peptide (titrant) were prepared by exchanging each into buffer containing 20 mM Tris, pH 7.4, 100 mM KCl, and 1 mM CaCl_2_. The concentrations of the IQ peptides (WT, Y1649A, I1654A, or F1658A) were each 10 μM in 1.5 ml in the sample cell for titration with 0.1 mM Ca_2_/CaM_12’_ and the concentrations of Y1657D and F1658D were each 50 μM in 1.5 ml for titration with 0.5 mM Ca_2_/CaM_12’_ using 35 injections of 10 μl each.

### NMR spectroscopy

All NMR measurements were performed at 303 K using a Bruker Avance III 600 MHz spectrometer equipped with a four-channel interface and triple-resonance cryoprobe. NMR sample preparation of Ca_2_/CaM_12’_-IQ was described previously (21). Two-dimensional NMR experiments (heteronuclear single quantum coherence [HSQC] and HSQC-IPAP) were recorded on samples of ^15^N-labeled Ca_2_/CaM_12’_ (0.5 mM) bound to unlabeled IQ (0.75 mM). Each sample was dissolved in 20 mM 2-Amino−2−hydroxymethyl-propane-1,3-diol-d_11_ (Tris-d_11_ at pH 7.5), 1.0 mM CaCl_2_, and 95% H_2_O/5% D_2_O. Three-dimensional NMR experiments for assigning backbone and side-chain resonances, and NOESY distance restraints were analyzed as described previously (42). NMR data were processed using NMRPipe (43) and analyzed with SPARKY (Goddard T.D. and Kneller D.G., University of California at San Francisco). To measure RDCs (23) of Ca_2_/CaM_12’_ bound to the IQ peptide, the filamentous bacteriophage Pf1 (Asla Biotech Ltd) was used as an orienting medium. Pf1 (12′ mg/ml) was added to an NMR sample that contained either ^15^N-labeled Ca_2_/CaM_12’_ bound to unlabeled IQ. ^1^H-^15^N residual dipolar coupling constants (D_NH_) were measured using a 2D IPAP (inphase/antiphase) ^1^H-^15^N HSQC experiment as described by (44). Representative IPAP-HSQC spectra of ^15^N-labeled Ca_2_/CaM_12’_ bound to the IQ peptide are shown in Fig. S4*A*. Briefly, the backbone N-H RDCs were calculated by measuring the difference in ^15^N splitting for each amide resonance, both in the presence and absence of the orienting medium. The RDC Q-factor and analysis of RDC data were calculated by PALES (45). The Q-factor is calculated as Q = RMS(D_meas_-D_calc_)/RMS(D_meas_), where D_meas_ is the measured RDC, D_calc_ is the calculated RDC, and RMS is the root mean square difference. A Q-factor of 30% corresponds to 2 Å resolution.

### NMR structure calculation

NMR-derived structures of Ca_2_/CaM_12’_ bound to the IQ peptide were calculated using restrained molecular dynamics simulations within Xplor-NIH (46). RDCs, NOE distances, dihedral angles from TALOS+ (47), and backbone hydrogen bonds were used as structural restraints. NOEs were obtained from ^15^N-edited NOESY-HSQC, ^13^C-edited NOESY-HSQC (aliphatic), and ^13^C-filtered NOESY-HSQC as described by (48). Representative ^13^C-edited NOESY-HSQC and ^13^C-filtered NOESY-HSQC spectra of ^13^C-labeled Ca_2_/CaM_12’_ bound to unlabeled IQ peptide are shown in Fig. S4, *B* and *C*, respectively. Backbone dihedral angles were calculated by TALOS+ (47) using backbone chemical shifts (H_α_, C_α_, C_β_, CO, ^15^N, and HN) as input. Hydrogen bond restraints in helices and β-sheets were verified by measuring amide hydrogen-deuterium exchange rates as described by (49). The Xplor-NIH structure calculation was performed in three stages: annealing, refinement, and water refinement (50). Annealing started from an extended random structure. A total of 200 structures were calculated and the one with lowest energy was used as a starting structure during the refinement. The lowest energy structure was refined in an explicit water environment. A Ramachandran plot was generated by PROCHECK-NMR (27) and structure quality was assessed by MolProbity (51).

### FP assays

Fluorescein-labeled peptides (100 nM; ChinaPeptides) were titrated with increasing concentrations of purified Ca_2_/CaM_12’_ in FP buffer (20 mM Tris, pH 7.4, 100 mM KCl, 1 mM MgCl_2_, 1.0 mM CaCl_2_) or apoCaM in Ca^2+^-free buffer (20 mM Tris, pH 7.4, 100 mM KCl, 1 mM MgCl_2_, 2.0 mM EGTA). FP was measured with a Synergy 2 plate reader (BioTek) as described (52). FP was calculated as P = (I_v_ - g∗I_h_)/(I_v_ + g∗I_h_); I_v_ and I_h_ are vertical and horizontal fluorescence intensity, respectively, and g is the correction factor for fluorescein. To obtain binding curves and *K*_*D*_ values, data were fitted in GraphPad Prism 5 (GraphPad Software Inc) to the equation Y = B∗X/(K_d_ + X); B is maximal FP value that would be reached at saturation as determined by extrapolation of the fitted curve.

### Concentration profiles of CaM species *versus* [Ca^2+^]

**Figure 8:**
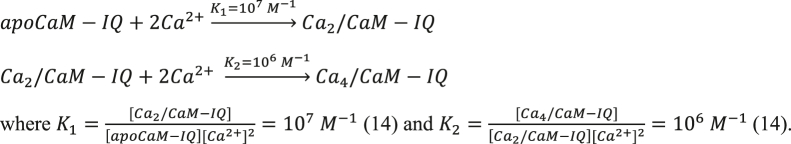
**Kinetic scheme for the sequential binding of Ca**^**2+**^**to the CaM C-lobe (K**_**1**_**) and CaM N-lobe (K**_**2**_**).**

The concentration profiles of apoCaM-IQ, Ca_2_/CaM-IQ, and Ca_4_/CaM-IQ as a function of the free Ca^2+^ concentration were calculated according to the following scheme in Figure 8.

### Expression of Ca_V_1.2 IQ domain mutants and CaM species in HEK 293T/17 cells

HEK 293T/17 cells (ATCC) were maintained as previously described (15, 53). For electrophysiology, Lipofectamine 2000 (Invitrogene) or JetPrime (Polyplus Transfection) was used to transiently transfect cells with indicated plasmid DNAs in 35 mm dishes. For biochemistry experiments, transient transfection of HEK 293T/17 cells in 100 mM dishes was achieved using either JetPrime or, as previously described (15, 53), the calcium phosphate method. Cells were cotransfected with plasmids encoding the pore-forming α_1_1.2 subunit N-terminally tagged with eCFP (15, 53) or mCherry (54) plus pGWIH-based plasmids encoding the auxiliary subunits rat β_2A_ (55) and rabbit α_2_δ-1 (56) as previously described (15, 53). For all transfections, equimolar ratio of 1:1:1 was used for Ca_V_1.2 channel subunits and later further optimized (JetPrime) for CaM (at ratio of 1:1:1:0.5 for α_1_1.2:β_2A_:α_2_δ−1:CaM). Rat brain α_1_1.2 (GenBank ID: M67515.1) N-terminally fused to eCFP was utilized as previously described (15). The point mutations in plasmids encoding single-residue I1654A, Y1657D (this report), and K1662E exchanges in α_1_1.2 were generated *via* QuikChange II as previously described (15, 53) using N-terminally eCFP (15, 53) or mCherry tagged (54) rat brain α_1_1.2 plasmid template DNAs. We studied CDI using mCherry-tagged α_1_1.2 subunit coexpressed with the other, untagged Ca_V_1.2 subunits and WT CaM or the calmodulin 34 mutant CaM_34_ (kindly provided by JP Adelman, (8)). For some biochemical experiments shown in Fig. S2, YFP-tagged CaM was used (32).

### Whole-cell patch clamp recording

Macroscopic Ba^2+^- (I_Ba_) and Ca^2+^ currents (I_Ca_) of Ca_V_1.2 L-type Ca^2+^ channels were obtained in the whole-cell configuration using external bath solution containing (in mM) 134 N-methyl-D-glucamine, 10 BaCl_2_ (for CDI, 10 CaCl_2_), 1 MgCl_2_, 10 Hepes, and 10 glucose with an adjusted pH of 7.4 (Cs-OH) and an osmolarity of 300 to 310 mOsm (sucrose). Intracellular pipette solution contained (in mM) 125 Cs-MeSO_3_, 5 CsCl, 10 EGTA, 10 Hepes, 1 MgCl_2_, 4 Mg-ATP, and pH 7.3 (CsOH), mOsm 290 to 300 (sucrose). Cells were clamped at a holding potential of -80 mV and depolarized for 900 ms to a series of activating potentials, from −60 mV to +50 mV (or +80 mV for Ca^2+^ currents), in increments of 10 mV at an interval of 0.033 Hz. The series resistance and the cell capacitance were directly taken from the Amplifier (Axopatch 200B, Molecular Device) and compensated to ∼40%. Data were sampled at 10 kHz and lowpass filtered at 2 kHz. Leak subtracted raw data were analyzed with Pclamp10 and GraphPad Prism IX software. All recordings were performed at room temperature (RT).

### Cell-attached patch clamp recording

Single-channel recordings were performed as described previously (15, 31). In brief, low noise raw data were recorded with an Axopatch 200B amplifier and data were sampled at 10 kHz with a low-pass filter at 2 kHz (3 dB, four pole Bessel) and digitalized with a Digidata 1440 digitizer. Recording electrodes were pulled from borosilicate capillary glass (0.86 OD/1.25 ID) with a Flaming/Brown micropipette puller (Model P-97, Sutter Instruments), heat polished, and coated with Sylgard (Sylgard 289) until close to the electrode tip. Electrode resistance in solution was usually 5 to 10 MΩ. To keep the membrane potential close to 0 mV the extracellular bath solution contained (in mM) 120 K-Glutamate, 25 KCl, 2 MgCl_2_, 1 CaCl_2_, 10 EGTA, 10 Hepes, and 2 Na_2_-ATP pH 7.4 (KOH). The intracellular pipette solution contained (in mM) 110 BaCl_2_ and 10 Hepes, adjusted to pH 7.4 (TEA-OH). Cells were depolarized for 2 s from a holding potential of -80 mV to 0 mV every 7 s. Event lists were created from raw Ba^2+^ currents after leak and capacity transients were digitally subtracted by pClamp 10. Unitary current events were then analyzed based on the half-height criterium (57) using the single-channel software provided by pClamp 10.

For statistical analysis, single-channel parameters were corrected by the channel number (k), respectively, the maximum of simultaneously open channels (P_MAX_). The number of channels in the patch was estimated based on the observed simultaneous openings and is a precise parameter for k < 4, as included in this article and originally described by R. Horn (58). On average, 100 to 200 Ba^2+^ current traces were recorded for each cell for each experimental condition for an appropriate statistical analysis.

### Surface biotinylation, NeutrAvidin pull downs, and immunoblotting

Surface biotinylation and analysis of Ca_V_1.2 surface expression was carried out essentially as described (15, 53) with the following modifications. Twenty-two to twenty-four hours post transfection, HEK 293T/17 cells plated in 100 mm diameter dishes were rinsed with RT PBS-CM (PBS supplemented with 1 mM Ca^2+^ and 0.5 mM Mg^2+^) and placed on ice. Cell were incubated with freshly prepared 0.4 mg/ml of EZ-Link-Sulfo-NHS-LC-biotin (Thermo Fisher Scientific) in PBS-CM for 30 min, followed by quenching of remaining NHS reactive groups with ice-cold 100 mM glycine in PBS-CM, four separate washes with quenching buffer, and a final rinse with PBS alone. Labeled and quenched cells were dislodged by scrapping and directly lysed into ice-cold radioimmunoprecipitation assay buffer (50 mM Tris-HCl, pH 7.4, 150 mM NaCl, 5 mM EGTA, 10 mM EDTA, 1% NP−40, 0.05% SDS, 0.4% DOC, and 10% glycerol) supplemented with protease inhibitors: 1 μg/ml leupeptin (Merck Millipore), 2 μg/ml aprotinin (Merck Millipore), 1 μg/ml pepstatin A (Merck Millipore), and 34 μg/ml PMSF (Sigma). Lysates were cleared of insoluble material *via* centrifugation at 200,000*g* for 30 min at 4 °C. The protein concentration of the solubilized material in the cleared lysate was determined by a standard bicinchoninic acid assay (Thermo Fisher Scientific). Biotinylated constituents in equal amount protein lysates (*e*.*g*., 400 μg/sample) were affinity purified by incubation with 30 μl of NeutrAvidin-conjugated Sepharose beads (Thermo Fisher Scientific) for 2 h at 4 °C. Bead-bound material was sedimented by centrifugation, washed several times with ice-cold buffer, and bound proteins extracted in SDS sample buffer (with shaking at 65 °C for 15 min). Proteins from pull downs as well as directly loaded lysates were fractionated by 7.5% acrylamide SDS–PAGE and transferred onto polyvinylidene difluoride (PVDF; Bio-Rad) membranes. For experiments used for analysis of CaM expression levels in directly loaded lysates (Fig. 6), 12% acrylamide gels were used. PVDF membranes were stained with Ponceau S, imaged, washed, and then incubated in blocking buffer (150 mM NaCl, 10 mM Tris–HCl, pH 7.4 (TBS) with 0.1% Tween (TBST) and 2% bovine serum albumin (RPI Corp.)) for 1 h at RT and then incubated with primary antibodies in blocking buffer for 3 h at RT. For analysis of surface expressed Ca_V_1.2, α_1_1.2 was detected using rabbit antibodies against epitopes in the intracellular loop II/III (FP1 or CNC1) and the CNC2 epitope near the C terminus of α_1_1.2 (59). When CaM expression in directly loaded lysates was assessed, the membranes were probed with a mouse anti-CaM monoclonal primary antibody (made against a synthetic peptide corresponding to the 21 carboxy terminal amino acids (128–148) of bovine calmodulin) obtained from from Sigma Millipore (catalog no.: # 05-173, Lot # 2717626). YFP-tagged CaM signals were further verified by the NeuroMab mouse anti-GFP monoclonal antibody N86/8 (UC Davis). Signals obtained from probing with antibodies against the cytosolic proteins GAPDH (mouse monoclonal, Sigma/Millipore 214592) and α-tubulin (DM1A mouse monoclonal, Santa Cruz Biotechnology SC32293) were used (along with Ponceau S-stained bands) as loading controls for correction of variation in protein content between lysate samples. The absence of GAPDH and α-tubulin antibody signals in NeutrAvidin-pull down samples also served as intracellular protein controls for assurance of plasma membrane integrity during the biotinylation of plated cells. PVDF membranes were washed for 40 min with at least five exchanges of TBST, incubated with horseradish peroxidase–conjugated secondary goat antimouse antibodies (Jackson) or mouse anti-rabbit antibodies (Jackson) for 1 h at RT, and washed again with TBST with at least five exchanges for 1.5 h. Immunosignals were detected using the horseradish peroxidase substrates Luminata Classico or Crescendo (Merck Millipore) or Femto (Thermo Fisher Scientific) by X-ray film (Denville Scientific Inc). Multiple exposures over increasing time periods were taken to ensure that all signals were in the linear range (60, 61).

### Analysis of immunoblots

Signal intensity for each band in scanned film images of immunoblots were assessed using ImageJ (https://imagej.nih.gov). Background signals in individual lanes were subtracted from the band signal prior to quantitative analysis. Differences in immunosignal strengths were corrected for potential immunoblotting and film exposures differences between experiments, as described (15, 53). Loading control (*e.g*., GAPDH, α-tubulin) lysate immunosignals were used to correct for minor differences in protein amounts loaded in individual sample lanes. To correct for variation in test immunosignals (*e.g*., α_1_1.2, CaM) between experimental replicates, normalization was done according to the ‘sum of the replicates’ method as described (62). Each immunosignal for a protein (*e.g*., α_1_1.2, CaM) on one blot was divided by the sum of all immunosignals from the same immunoblot exposure for that experimental run to obtain the relative signal fraction for each band (62). The means of these signal intensity fractions were calculated for each condition (*e.g*., α_1_1.2 WT, Y1657D) from all experiments (*e.g*., α_1_1.2 WT, Y1657D) and these means then divided by the mean value of the test control (*e.g*., α_1_1.2 WT, which is now equal to 1% or 100%). All data were statistically analyzed (GraphPad Prism IX software) applying either a Student's *t* test (two-sample comparison) or ANOVA with Tukey post hoc test.

## Data availability

Atomic coordinates were deposited in the Protein Databank (accession no. 7L8V), and all other data are contained within the article.

## Supporting information

This article contains supporting information (13, 19, 28, 63).

## Conflict of interest

The authors declare that they have no conflicts of interest with the contents of this article.
