# Supplementary material for: Half-calcified calmodulin promotes basal activity and inactivation of the L-type calcium channel CaV1.2
Source: J Biol Chem. 2022 Nov 15;298(12):102701. doi: 10.1016/j.jbc.2022.102701 (PMC9764201; doi:10.1016/j.jbc.2022.102701)
Supplement: Supporting information [file mmc1.pdf]

Supporting Information

Half-calcified calmodulin promotes basal activity and inactivation of the L-type calcium channel $Ca_V1.2$.

Peter Bartels[1#], Ian Salveson[2#], Andrea M. Coleman[1,2#], David E. Anderson[2#], Grace Jeng[1], Zoila M. Estrada-Tobar[1], Kwun Nok Mimi Man[1], Qinhong Yu[2], Elza Kuzmenkina[3], Madeline Nieves-Cintron[1], Manuel F. Navedo[1], Mary C. Horne[1*], Johannes W. Hell[1*] and James B. Ames[2*]

[1]Department of Pharmacology, University of California, Davis, CA 95616, USA.

[2]Department of Chemistry, University of California, Davis, CA 95616, USA.

[3]Center for Pharmacology, University of Cologne, Cologne, Germany.

[#]PB, IS, AMC and DEA contributed equally to this work.

[*]To whom correspondence should be addressed: (MCH) Department of Pharmacology, University of California at Davis, Davis CA 95616. Telephone: 530-752-7723; E-mail: mhorne@ucdavis.edu. (JWH) Department of Pharmacology, University of California at Davis, Davis CA 95616. Telephone: 530-752-6540; E-mail: jwhell@ucdavis.edu. (JBA) Department of Chemistry, One Shields Avenue, University of California, Davis, CA 95616, Tel (530) 752-6358, FAX (530) 752-8995, email: jbames@ucdavis.edu.

The Supporting information includes:
Figures S1 to S4
Tables S1 to S4
SI References

Supplementary Figure 1

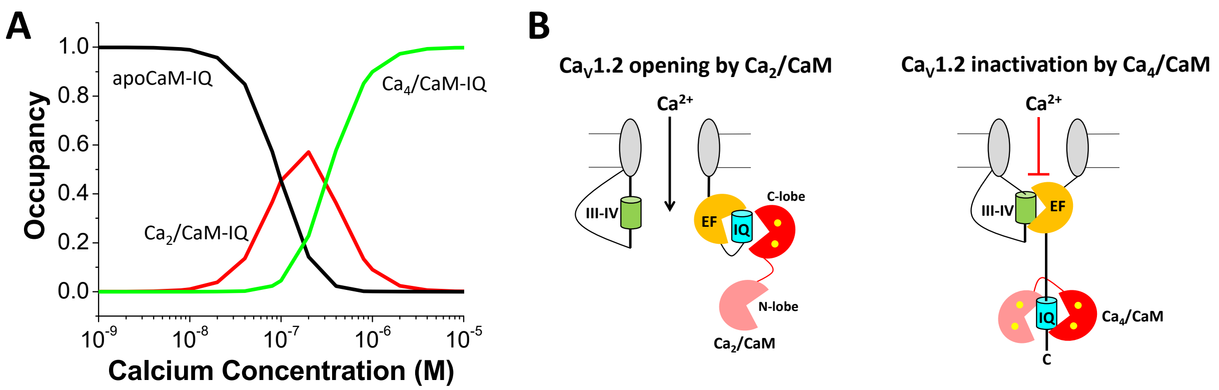

Fig. S1. Occupancy of IQ bound CaM species vs [Ca^{2+}] and Schematic Model. (**A**) Concentration profiles of apoCaM bound to IQ (apoCaM-IQ, black), half Ca^{2+}-saturated CaM (Ca_2/CaM-IQ, red line) and Ca^{2+}-saturated CaM (Ca_4/CaM-IQ, green line). The concentration profiles were calculated as described in Materials and Methods. (**B**) Schematic model of Cav1.2 channel regulation by CaM modified from (1). Cav1.2 channel opening facilitated by half-calcified Ca_2/CaM (left panel) and channel inactivation by fully calcified Ca_4/CaM (right panel): channel domain (gray), EF-hand domain (orange), IQ (cyan), III-IV linker (green), CaM (red), bound Ca^{2+} (yellow). This schematic model integrates earlier data with the recent cryoEM structure of the related Cav1.1 channel (2) and with our current work. The Cav1.1 cryoEM structure likely represents the inactivated state of the channel because the net potential is nominally 0. In the cryoEM structure, the EF-hand region is in close proximity to the loop between domains III and IV. The binding of the channel EF-hand domain with the III-IV helix resembles a "plug" near the entrance of the channel that may restrict Ca^{2+} influx through the inactivated channel at high Ca^{2+} levels that cause CDI. The III-IV helix interaction with the channel EF-hand is structurally similar to the CaM N-lobe (EF1 and EF2) bound to the IQ-helix seen in the CaM-IQ crystal structure (3). We propose that under basal conditions ([Ca^{2+}] = 100 nM) the channel EF-hand domain binds to one side of the IQ helix (stabilized by EF3 and EF4 of CaM bound on the opposite side), which keeps the channel EF-hand away from loop III/IV to clear a path for Ca^{2+} entry (marked by an arrow in panel B). Upon Ca^{2+} influx, the Ca^{2+}-bound EF1 and EF2 of CaM will now bind to IQ and displace the EF-hands of Cav1.2, which allows these EF-hands to bind to the III/IV loop. This model is consistent with data presented by (4), who state: "C-lobe CDI likely requires a tripartite complex of IQ, PCI, and C-lobe Ca/CaM" and that the I/A and Q/A mutants "actually demonstrated strong and graded reductions in affinity" for the EF-hand and IQ domain interaction "coarsely matching observed deficits in C-lobe CDI (Fig. 6B)." However, their data do not distinguish whether formation of such a tripartide complex occurs under basal conditions when a full length Ca^{2+} channel is present, or, as they propose, upon Ca^{2+} influx through activated channel. Their structural model with the CaM C-lobe attached to the channel EF-hands and the calcified CaM N-lobe bound to the NSCaTE segment (present in one but not the other splice variant of the N-terminus of Cav1.2) seems less likely because this model would prevent the channel EF-hand from interacting with loop III/IV during CDI as seen in the cryoEM structure of Cav1.1 (2). According to our model, under basal conditions Ca_2/CaM binds to one face of the IQ motif and then the upstream EF-hands, which constitute most of this PCI region, can bind to the other face. This interaction likely requires the presence of CaM because the IQ motif is unstructured in the

highly homologous Cav1.1 region but the addition of CaM induces formation of the alpha-helix (2). Notably, deficits in CDI and binding interactions of mutants in the EF region and also in the IQ motif were overcome by overexpression of CaM in (4), again consistent with our model.

Supplementary Figure 2

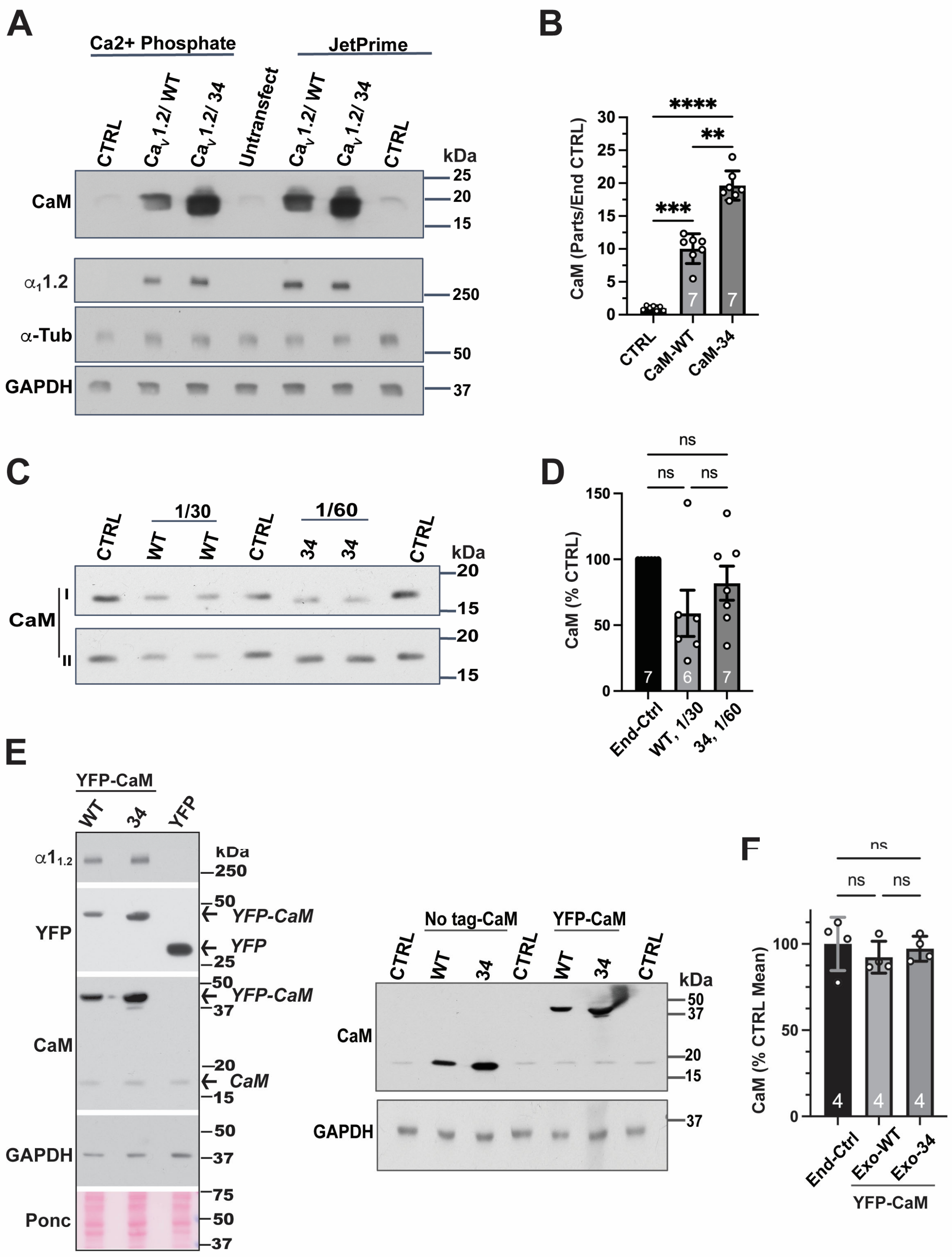

Fig. S2. Ectopic expression of WT CaM and CaM$_{34}$. (**A,B**) Immunoblots of total lysates of HEK 293T/17 cells transfected with $\alpha_1$1.2, $\alpha_2\delta$1, and β_{2A} plus untagged (pJPA7) WT CaM or CaM$_{34}$ using calcium phosphate versus JetPrime or mock-transfected (CTRL) or untransfected HEK 293T/17 cells. Tubulin (α-Tub) and GAPDH were used as internal loading controls. (**B**) Quantification of CaM immunosignals normalized to endogenous CaM (set to value of 1) as detected in mock-transfected CTRL cells (n=7 independent experiments; one-way ANOVA (F=174.8, p<0.0001), Tukey post-hoc test, **p<0.01, ***p<0.001, and ****p<0.0001). (**C,D**) Immunoblots of diluted lysates of WT CaM (30-fold dilution) and CaM$_{34}$ (60-fold dilution) transfected cells from two different experiments. (**D**) Quantification of CaM immunosignals normalized to endogenous CaM signal (set to 100%) as detected in mock-transfected CTRL cells (WT CaM, n = 6; CaM$_{34}$, n = 7; one-way ANOVA (F=2.844, p=0.0860), Tukey post-hoc test, ns=p > 0.05). (**E,F**) Immunoblots of total lysates of cells transfected with untagged or YFP-tagged WT CaM or CaM$_{34}$ using calcium phosphate or mock transfected (CTRL) HEK 293T/17 cells. Anti-CaM probings indicate expression level of CaM$_{34}$ muant (untagged and YFP-tagged) relative to overexpressed and endogenous WT CaM. Anti-GFP immunoblotting verifies anti-CaM signal intensities in lysates from YFP-tagged WT and CaM$_{34}$ mutant calmodulin (left blot in **E**), while GAPDH and Ponceau S staining (Ponc) was used as loading controls. (**F**) Quantification of immunosignals at 17 kDa reflecting endogenous (Endo) CaM in YFP-tagged CaM transfected samples normalized to mean (set to 100%) of endogenous CaM levels (CTRL); n = 4; one-way ANOVA (F= 0.4856, p=0.636), Tukey post-hoc test, ns=p > 0.05). Signals for YFP-CaM and untagged CaM in the respective cDNA-transfected cell lysates are not quantified here.

Supplementary Figure 3

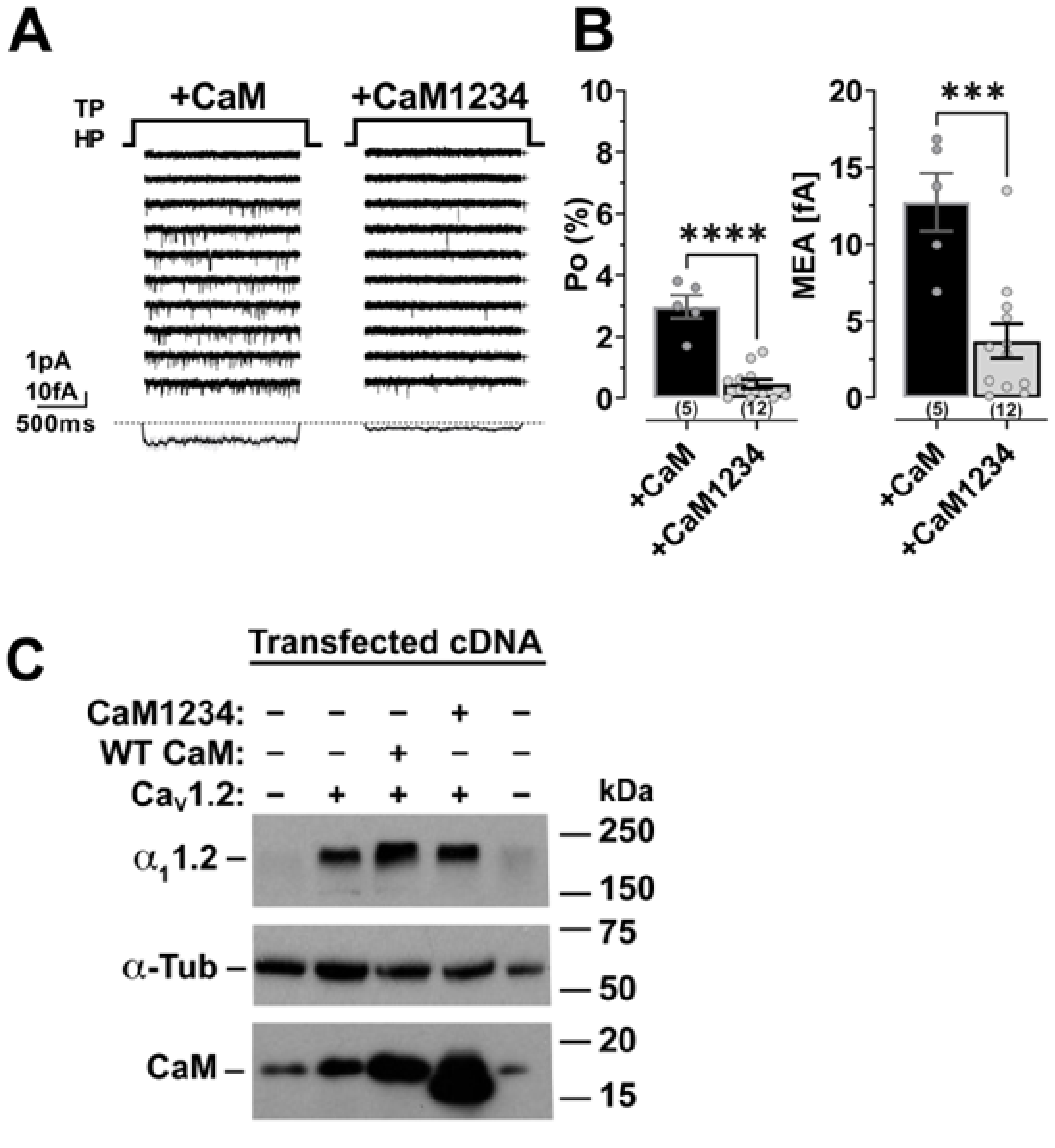

Fig. S3. Effects on ectopic expression of WT CaM and CaM_{1234} on $Ca_V1.2$ activation and protein expression. HEK 293T/17 cells were transfected with $\alpha_1 1.2$, $\alpha_2\delta 1$, and β_{2A} plus WT CaM or CaM_{1234}. (**A**) 10 consecutive representative single-channel traces of WT Cav1.2 expressed with WT CaM (left) or CaM_{1234} (right). Bottom: MEA calculated from a total of 810 superimposed traces for Cav1.2 expressed without CaM (n=5 cells) and 1952 traces for Cav1.2 expressed with CaM_{1234} (n=12 cells). (**B**) Quantification of Po (left) and MEA (right) reveal a robust decrease in channel activity for ectopic expression of CaM_{1234} compared to CaM. Numbers in parenthesis under bars reflect n independent recordings (***p<0.001 and ****p<0.0001, unpaired, two-tailed Student's T-test. (**C**) Immunoblots of lysates from HEK 293T/17 cells transfected with (+) or without (-) the indicated expression vector DNAs. The blotted membranes were probed with the antibodies indicated at the left side of each membrane piece spanning the indicated molecular weight ranges at the right.

Supplementary Figure 4

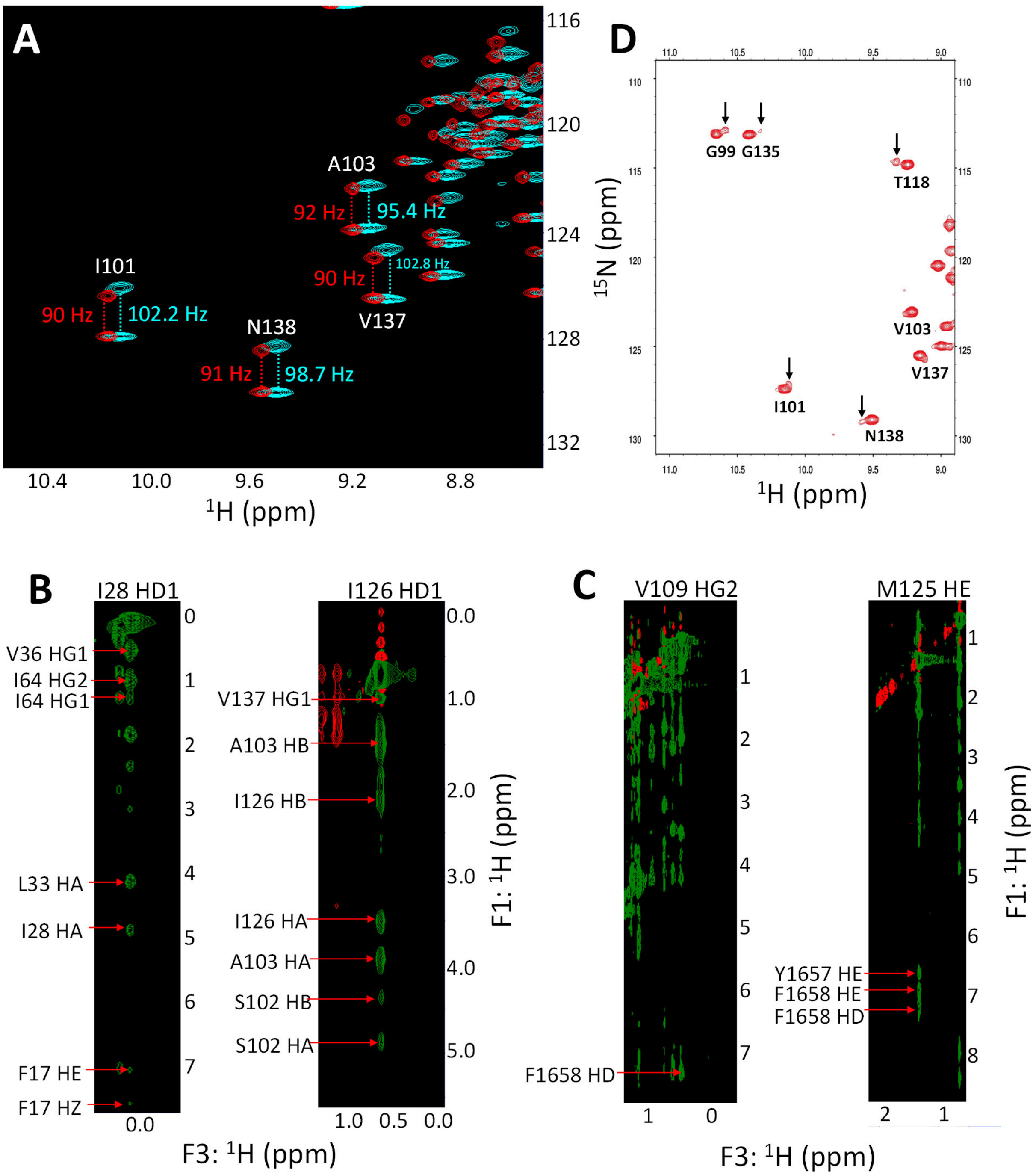

Fig. S4. NMR-derived structural restraints for Ca_2/CaM$_{12'}$-IQ complex. (A) Representative IPAP-HSQC spectrum of ^{15}N-labeled Ca_2/CaM$_{12'}$ bound to unlabeled IQ peptide. Samples were prepared by adding five milligrams of ^{15}N-labeled Ca_2/CaM$_{12'}$-IQ complex to 0.5 mL of NMR buffer (see Methods) containing 12 mg/mL of filamentous bacteriophage Pf1. IPAP-HSQC spectra were recorded in the presence of Pf1 (cyan peaks in A) and absence of Pf1 (red peaks in A). Residual dipolar couplings (RDCs) were measured (in Hertz) as the difference in splitting for the ^{15}N-[^1H] doublet components relative to the isotropic $^1J_{NH}$ coupling. RDCs measured for 24 residues served as orientational structural restraints applied during the refinement phase of the

structure calculation. A complete list of RDCs (derived from IPAP-HSQC) and distance restraints (derived from the NOESY spectra) is provided in PDB entry 7L8V. **(B)** Strip plot analysis of three-dimensional ^{13}C-edited NOESY-HSQC and **(C)** ^{13}C-filtered NOESY-HSQC spectra of ^{13}C-labeled $Ca_2/CaM_{12'}$ bound to unlabeled IQ peptide. Assigned crosspeaks (marked by residue labels) in the NOESY spectra indicate a correlation between two residues that are in close proximity (< 5 Å apart). (D) HSQC spectrum of ^{15}N-labeled $Ca_2/CaM_{12'}$ (100 μM) bound to unlabled IQ peptide (100 μM) shows sample heterogeneity. The experimental conditions and further details are provided in the Experimental Procedures.

Supplementary Tables

Supplementary Table 1

Table S1. HEK 293T/17 cells were transiently transfected with eCFP-tagged rat Cav1.2 (WT versus I1654A and K1662E or versus Y1657D) plus $\alpha_2\delta1$ and β_{2a} and whole cell current recorded (see Fig. 3 for more details).
Panel A. Peak current density from I/V curves of whole-cell recordings. Imax values were determined at +20 mV for Ca^{2+} and 0 mV for Ba^{2+}.
Panel B. r300 values and quantification of CDI and VDI. Whole-cell currents remaining after 300 ms of depolarization (r_{300}) are given as fraction of maximal currents based on values in **A**. Net CDI was calculated as the ratiometric value net $1-(Ca_{300}/Ca_{max})/(Ba_{300}/Ba_{max})$ and VDI as $1-(Ba_{300}/Ba_{max})$.
For each panel: **Top:** Analysis of interleaved recordings of Cav1.2 WT versus I1654A and K1662E IQ- mutants (one-way ANOVA with a Bonferroni post-hoc test; $^{*}p<0.05$, $^{**}p<0.01$ and $^{***}p<0.001$ and $^{****}p<0.0001$). **Bottom:** Analysis of interleaved recordings of Cav1.2 WT versus Y1657D IQ-mutant Cav1.2 (unpaired T-Test, $^{*}p<0.05$ and $^{****}p<0.0001$). All data show as mean±SEM and numbers in parentheses (n) are independent experiments.

A.

IQ-variant	$ImaxCa^{2+}$(pA/pF)	$ImaxBa^{2+}$(pA/pF)	p-value
**Ca$_V$1.2**	18.3±4.2(10)	46.7±9.1(11)	n/a
**I1654A**	**2.5±0.8(8)	*19.2±5.6(9)	
**K1662E**	10.9±3.2(10)	39.1±6.6(12)	>0.2/>0.9
**Ca$_V$1.2**	16.4±2.1(13)	21.8±4(11)	n/a
**Y1657D**	***5.8±1(11)	*11.8±2.2(11)	

B.

IQ-variant	$ImaxCa^{2+}(r_{300})$	$ImaxBa^{2+}(r_{300})$	$^{\dagger}netCDI_{300}$	$^{\#}VDI_{300}$	p-value $netCDI_{300}/VDI_{300}$
**Ca$_V$1.2**	0.33±0.03(10)	0.81±0.02(10)	0.59±0.04(10)	0.19±0.02(10)	n/a
**I1654A**	0.53±0.05(8)	0.71±0.04(8)	****0.23±0.06(8)	*0.30±0.03(8)	
**K1662E**	0.40±0.02(10)	0.85±0.03(10)	0.52±0.04(10)	0.16±0.03(10)	†>0.56/$^{\#}$>0.77
**Ca$_V$1.2**	0.32±0.03(11)	0.83±0.02(11)	0.61±0.03(11)	0.17±0.02(11)	n/a
**Y1657D**	0.59±0.03(10)	0.76±0.05(10)	****0.21±0.05(10)	0.25±0.05(10)	†<0.0001/$^{\#}$>0.12

Supplementary Table 2

Table S2. Biophysical properties of WT and Y1657D mutant $Ca_V1.2$ as determined by single channel recordings with 110 mM Ba^{2+}. HEK 293T/17 cells were transiently transfected with eCFP-tagged rat $Ca_V1.2$ (WT versus Y1657D) plus $\alpha_2\delta1$ and β_{2a}. Single-channel parameter were corrected for the number of channels (*k*) in the patch. Availability denotes the number of active over passive traces. Popen (Po) is the open probability within active traces. Ipeak is the current flown through all channels in a patch at the time point of the current maximum and was corrected by the number of channels. Mean ensemble average (MEA) is the mean average current of all traces after being superimposed. The mean open time (MOT) is the arithmetic mean value of the channel open time. The unitary current amplitude (i) was measured at the test potential of 0 mV applied from the holding potential of -80 mV. An unpaired T-test was applied for all parameters to determine the p values. All data show mean±SEM and numbers in parentheses (n) numbers of independent experiments.

**Parameter**	**$Ca_V1.2$ (8)**	**Y1657D (8)**	**p-value**
Availability (%)	65±12	34±7	<0.05
Po (%)	2.0±0.5	0.34±0.1	<0.01
I_{Peak} [fA]	21.2±5.3	7.4±1.3	<0.01
MEA [fA]	8.5±2.4	2.0±0.6	<0.05
MOT (ms)	1.4±0.2	1.2±0.2	>0.05
unitary i (pA)	0.7±0.04	0.7±0.02	>0.05
number of traces	857	1366	

Supplementary Table 3

Table S3. Effects of ectopically expressed CaM and CaM_{34} on $Ca_V1.2$ as determined by single channel recordings with 110 mM Ba^{2+}. HEK 293T/17 cells were transiently transfected with eCFP-tagged rat $Ca_V1.2$, $\alpha_2\delta1$ and β_{2a} alone or together with WT CaM (+CaM) or CaM_{34} (+CaM_{34}). Parameters were corrected for the number of channels (*k*) in the patch. Recordings were obtained at a test potential of 0 mV from a holding potential of -80 mV. For all parameters, statistical significance p was determined for $Ca_V1.2$ without ectopic CaM expression and for $Ca_V1.2$ plus CaM_{34} agains $Ca_V1.2$ plus wt CaM (one-way ANOVA followed by Bonferroni test). All data show mean±SEM and numbers in parentheses (n) numbers of independent experiments.

**Parameter**	**$Ca_V1.2$ (11) †**	**+CaM (12)**	**+CaM_{34} (9) #**	**p-value**
Availability (%)	77±7	82±8	84±6	†>0.9999/#>0.9999
Po (%)	2.3±0.6	6.1±1.5	0.9 ±0.2	†<0.05/#<0.01
I_{Peak} [fA]	19.7±4.0	41.9±8.9	12.7±1.1	†<0.05/#<0.01
MEA [fA]	9.6±2.4	24.8±6.5	4.9±0.8	†<0.05/#<0.01
MOT (ms)	1.2±0.2	1.2±0.1	1.2±0.1	†>0.9999/#>0.9999
unitary i (pA)	0.81±0.03	0.81±0.03	0.82±0.03	†>0.9999/#>0.9999
number of traces	2009	2327	1655	

Supplementary Table 4

Table S4. Effects of ectopically expressed CaM_{1234} on $Ca_V1.2$ as determined by single channel recordings with 110 mM Ba^{2+}. HEK 293T/17 cells were transiently transfected with eCFP-tagged rat $Ca_V1.2$, $\alpha_2\delta1$ and β_{2a} together with either WT CaM or CaM_{1234}. Parameters were corrected for the number of channels (k) in the patch. Recordings were obtained at a test potential of 0 mV from a holding potential HP of -80 mV. For all parameters, statistical significance p was determined for $Ca_V1.2$ plus CaM_{1234} agains $Ca_V1.2$ plus wt CaM (unpaired T-test). All data show mean±SEM and numbers in parentheses (n) numbers of independent experiments.

**Parameter**	**+CaM (5)**	**+CaM1234 (12)**	**p-value**
Availability (%)	75±9	46±8	p>0.0626
Po (%)	3.0±0.4	0.5±0.1	p<0.0001
I_{Peak} [fA]	26.4±4.6	9.5±1.2	p<0.001
MEA [fA]	12.7±1.9	3.7±1.1	p<0.001
MOT (ms)	1.2±0.1	1.2±0.1	p>0.7
unitary i (pA)	0.81±0.03	0.93±0.04	p>0.07
number of traces	810	1952	

SI References

1. J. B. Ames, L-Type Ca(2+) Channel Regulation by Calmodulin and CaBP1. *Biomolecules* **11**, 1811 (2021).
2. J. Wu *et al.*, Structure of the voltage-gated calcium channel Ca(v)1.1 at 3.6 A resolution. *Nature* **537**, 191-196 (2016).
3. F. Van Petegem, F. C. Chatelain, D. L. Minor, Jr., Insights into voltage-gated calcium channel regulation from the structure of the CaV1.2 IQ domain-Ca2+/calmodulin complex. *Nature structural & molecular biology* **12**, 1108-1115 (2005).
4. M. Ben Johny, P. S. Yang, H. Bazzazi, D. T. Yue, Dynamic switching of calmodulin interactions underlies Ca2+ regulation of CaV1.3 channels. *Nature communications* **4**, 1717 (2013).
